# Proteome Folding Kinetics Is Limited by Protein Halflife

**DOI:** 10.1371/journal.pone.0112701

**Published:** 2014-11-13

**Authors:** Taisong Zou, Nickolas Williams, S. Banu Ozkan, Kingshuk Ghosh

**Affiliations:** 1 Center for Biological Physics, Department of Physics, Arizona State University, Tempe, Arizona, United States of America; 2 Department of Physics and Astronomy, University of Denver, Denver, Colorado, United States of America; University of Leeds, United Kingdom

## Abstract

How heterogeneous are proteome folding timescales and what physical principles, if any, dictate its limits? We answer this by predicting copy number weighted folding speed distribution – using the native topology – for E.coli and Yeast proteome. E.coli and Yeast proteomes yield very similar distributions with average folding times of 100 milliseconds and 170 milliseconds, respectively. The topology-based folding time distribution is well described by a diffusion-drift mutation model on a flat-fitness landscape in free energy barrier between two boundaries: i) the lowest barrier height determined by the upper limit of folding speed and ii) the highest barrier height governed by the lower speed limit of folding. While the fastest time scale of the distribution is near the experimentally measured speed limit of 1 microsecond (typical of barrier-less folders), we find the slowest folding time to be around seconds (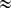
8 seconds for Yeast distribution), approximately an order of magnitude less than the fastest halflife (approximately 2 minutes) in the Yeast proteome. This separation of timescale implies even the fastest degrading protein will have moderately high (96%) probability of folding before degradation. The overall agreement with the flat-fitness landscape model further hints that proteome folding times did not undergo additional major selection pressures – to make proteins fold faster – other than the primary requirement to “sufficiently beat the clock” against its lifetime. Direct comparison between the predicted folding time and experimentally measured halflife further shows 99% of the proteome have a folding time less than their corresponding lifetime. These two findings together suggest that proteome folding kinetics may be bounded by protein halflife.

## Introduction

Diverse pool of protein sequences give rise to an astonishing degree of heterogeneity in the biophysical properties across the proteome. This raises a fundamental question: how heterogeneous is the proteome? Recent work showed biophysical properties have broad distributions across the proteome and their consequences at the phenotypic level [1]–[5]. While sequence variation alone would lead to such diverse biophysical properties, there are other features of the cellular environment – for example protein abundance, role of chaperones, co-translational folding – that can further influence these distributions. Protein copy number – although neglected in the earlier calculations of distributions – in particular can play a crucial role due to a possible correlation with biophysical properties such as folding stability [6]. It has been well established that highly abundant proteins are slowly mutating [7], [8]. The reason behind this negative correlation is believed to be the selection pressure against cytotoxicity of misfolded proteins arising due to lower stability. Rules of protein biophysics has been used to quantitatively establish the relation between abundance and stability [6], [8]. On the other hand, it is believed that there may be a possible correlation between stability and folding speed [9]–[11]. Thus, it is tempting to hypothesize that protein abundance and folding speed may be related as well. A natural question arises – how does protein abundance alter, if at all, the folding time distribution? Without *a priori* knowledge of the effect of protein abundance on the folding time distribution, it is imperative that any attempt to predict the folding time distribution of a proteome should consider the effect of abundance as well.

Learning about the extent of heterogeneity in biophysical properties across the proteome in itself is a fundamental question – leading further inquires on the details of the distribution. For example in case of folding time distribution, what are the lower and upper speed limits? What physical principle dictates these limits? What is the peak value, if any, of the distribution? Is there a limiting behavior due to competition with other time scales such as diffusion, protein synthesis, degradation? If kinetic stability [12] – introducing higher barrier height while keeping the same value for the free energy difference between the folded and the unfolded state – is a strategy cells use to minimize exposure to unfolded states to avoid lethal effects of aggregation or degradation [13], do we expect proteomes to be biased towards higher folding times? And if so, how do these timescales compare with protein halflife, in other words is the proteome folding timescale still able to beat the degradation clock with an increased barrier height? While outpacing degradation appears to be important, are there any other selection pressures that may have influenced proteome folding kinetics? Furthermore, how do these distributions vary across different kingdoms of life – for example between Escherichia coli (E.coli) and Yeast – or is there an universality in the shape of the distribution? In this article, we attempt to determine proteome folding kinetics distribution and address some of these fundamental questions.

## Materials and Methods

### Determining the folding speed of a protein

Plaxco, Simons, Baker [14] made the observation that relative contact order (CO), a metric based on the native topology of the protein, correlates well with the folding speed measured *in vitro*. CO is defined as the average residue separation – normalized by the chain length – of atomic contacts present in the native structure of the protein [14]. Since the pioneering work of Plaxco, Simons, Baker there have been numerous efforts to understand its implication [15] and establish the role of other native-centric metric [16]–[21] and their relative performances to predict the folding speed of proteins using native structure [18], [20], [21]. One such effort has shown absolute contact order (ACO) – defined as the product of CO and the chain length – predicts folding speeds more accurately than CO for bigger set of proteins [16]. In a nutshell, all these different metrics provide a prescription to predict the folding speed of a protein with the knowledge of the native structure alone. We utilize this powerful idea to predict the folding time distribution for proteins in the proteome for which the exact (or highly homologous) native structures are known. Recent work by Rustad and Ghosh [21] has provided a first principle explanation – employing polymer physics arguments – for the observed correlation between absolute contact order (ACO) [16] and folding speed. Furthermore, within a perturbative scheme, the work has proposed an extension of the metric (ACO) that captures the effect of different loop topologies [21]. This new metric, minor variation of ACO, provides slight improvement over ACO when benchmarked against the largest set (116 proteins) of *in vitro* folding speed data. We use this new modified metric, instead of ACO, to predict the folding speed from the native structure of the protein. For a given protein, we predict folding speeds for different domains, assuming each domain folds independently. Since the domain with the slowest folding speed is rate limiting, we use the folding speed of the slowest folding domain to be the folding speed of the protein.

### Curating the fraction of proteome that have both the structure and abundance data available

In order to predict folding speed, as described above, we need the information about the native structures of proteins in the proteome. We collect proteins from the Yeast and E.coli proteome for which the structures of proteins are available. For the Yeast proteome we use domain assignment from Yeast resource center (YRC) database [22]. Next we perform a BLAST search of the corresponding sequences to identify the best possible match for their structures. We list only those proteins that simultaneously satisfy a minimum of 80% sequence coverage and 50% identity match. In order to predict copy number weighted folding time distribution, we gather proteins for which both the structure and abundance information are available. We cross reference the curated list of proteins with available structure, described above, against the integrated list from PaxDB database [23]. The integrated list is the most comprehensive list of protein abundance values. We choose this list to ensure maximum coverage of proteins from the proteome. This method yields a total of 755 Yeast proteins. For E.coli proteome, we follow a similar approach but use the dataset collected by O′Brien *et al.* [24]. The original dataset reported in O′Brien *et al.* categorizes proteins (and their domains) based on a single abundance scale. We cross reference the combined list against the integrated list of abundance from PaxDb [23] yielding a total of 848 E.coli proteins. In summary, our datasets (Table S1 and S2) provide the largest fraction of proteomes (in E.coli and Yeast) for which both the abundance and structural informations are now available.

## Results and Discussion

### Folding time distribution is heterogeneous

**Figure 1:**
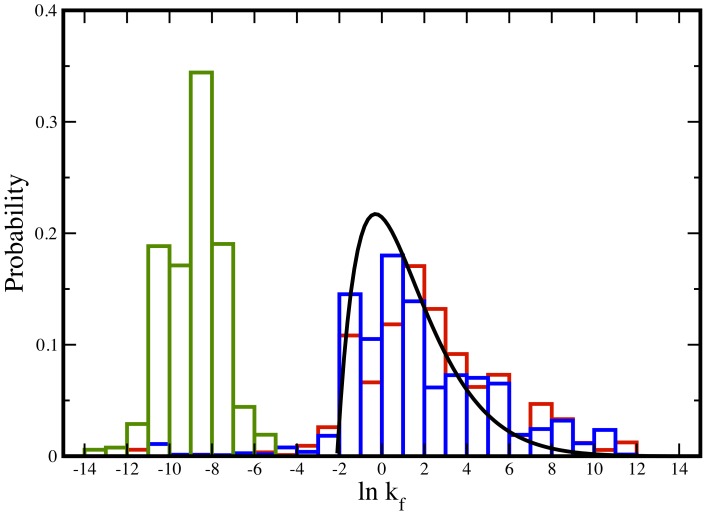
Folding speed (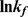) distribution – calculated using native topology – of E.coli (in red) and Yeast (in blue) weighted by protein copy number. The distribution of average lifetime for proteins in Yeast [27] is shown in green. The predicted folding time distribution using a diffusion-drift model (equation 5) with the boundary condition of the maximum folding time of 8 seconds is shown in black. Maximum folding time of 8 seconds was determined by best fitting Yeast distribution.

Copy number weighted folding speed (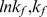 being the folding speed) distributions in E.coli and Yeast show a broad range of folding speeds, from several microseconds^−1^ to minutes^−1^ (Figure 1). The fastest folding time is in the neighborhood of microseconds. This is consistent with studies on ultrafast folding proteins defining the speed limit of protein folding [21], [25], [26]. It is interesting to note the lower speed limit is of the order of seconds to minutes, in proximity to the scale of halflives of short-lived proteins [27]. The implication of this observation will be discussed in detail in the section below. The average folding time (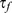) for copy number weighted distribution is calculated as
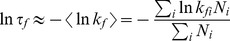
(1)where, 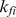 and 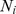 are the folding speed and the copy number, respectively, of the 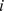 th protein. Average folding time without accounting for differential protein abundance levels can be obtained by simply setting 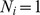. For E.coli, we find the average is approximately 100 milliseconds for copy number weighted distribution. The average remains almost unaltered when the distribution is not weighted by the protein expression level (i.e. setting 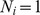, distribution not shown here). The average folding time for Yeast proteome is 170 milliseconds and 60 milliseconds for copy number weighted and unweighted distributions, respectively.

Recent work – grounded in the hypothesis of global selection against toxic effect of misfolding explaining observed correlation between abundance and evolution rate [8] – predicts highly abundant proteins are more stable [6]. Given this link between stability-abundance and *possible* interdependence between stability and folding kinetics [9]–[11], it is natural to expect a possible relation between abundance and folding speed as well. However, based on the results stated above, we do not see any noticeable effect of abundance on folding kinetics in E.coli. A possible explanation, among many other alternative ones, could be that the proteome can not afford to under-express slow folding proteins due to functional reasons. Furthermore, we notice a marginal slowing down of the proteome folding speed in Yeast upon weighting by protein abundance. Given the inherent uncertainties in predicting folding speed from native topology, a three-fold slowing down of the proteome is probably a very weak effect. However, if slowing down of the proteome due to copy number weighting is indeed beyond uncertainty, it may imply slow folding proteins are over-expressed for strong functional reasons despite the threat of misfolding. It may also imply the proteome is equipped with mechanisms such as chaperone-assisted folding, complex chaperone-substrate network [28] to mitigate possible deleterious effects of misfolding due to lower folding speed. As will be seen in later sections, three fold lowering of the speed around 60 millisecond timescale still allows proteins enough time to fold before degradation. It is interesting to note folding speed distributions in E.coli and Yeast – baring minor variations mentioned above – are very similar, indicating a universal behavior in the folding kinetics.

One caveat of our analysis is that the folding speed is predicted using models that have been benchmarked against *in vitro* folding data. However recent work, although limited, does not show significant differences between folding times measured *in vivo* and *in vitro* [29]. It is also important to note major conclusions remain the same if other metric such as ACO is used to predict the folding speed.

### Diffusion-drift model of mutations on a flat-fitness landscape explains the predicted distribution of folding speed

**Figure 2:**
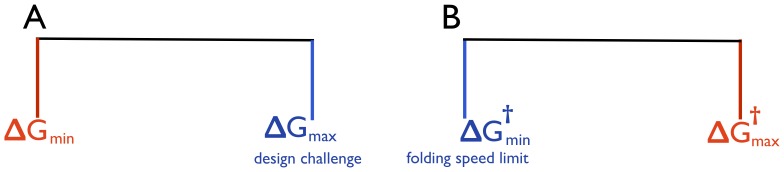
A) Accessible range in stability (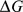 increasing towards right) is shown between blue and red lines. Black line shows the flat-fitness landscape for all values of stability greater than the minimum; i.e. 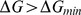, with the red line showing the drop in fitness when stability is lower than the minimum due to cytotoxic effects from aggregation/misfolding. Blue line shows the upper limit of stability (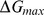) due to design challenge. B) Accessible range in the folding free energy barrier height (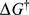 increasing to the right) between blue and red lines. Black line shows the flat-fitness landscape for all values of barrier heights less than the maximum allowed i.e. 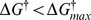, with the red line showing the compromised fitness when the barrier height is greater than the maximum leading to slow folding proteins, prone to aggregation and degradation. Blue line shows it is not possible to create proteins faster than the speed limit of folding set by barrier-less folders.

Apart from minor differences in details, the overall shape and the range of the distributions for E.coli and Yeast are roughly similar. The universal distribution (Figure 1) of the folding speed, irrespective of the details of the species, is well explained by a diffusion-drift model of mutations altering folding free energy barrier (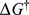). Shakhnovich *et al.* [1] used a similar model to describe a universal distribution of stability (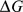). Due to close analogy between the two models, we briefly describe the stability model first. Further details of the model can be found in the work of Shakhnovich *et al.* [1]. Their model uses diffusion - arising from random mutations - with a drift to explain the stability distribution 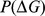. The model also imposes two boundary conditions 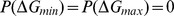 at the maximum (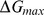) and minimum (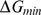) values of allowed stability. These two constraints can be explained as follows (Figure 2A): from design perspective, it is impossible to make proteins indefinitely stable, thus imposing an upper limit on the stability, hence 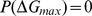. The boundary condition on the lower limit of stability, on the other hand, arises from the requirement of minimal stability to avoid misfolding that can be lethal to the phenotype of the organism. The model assumes a flat-fitness landscape for all values of stability greater than the minimum, i.e. 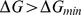. The fitness is severely compromised if stability drops below the threshold i.e. 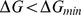, imposing the constraint 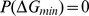. Thus, the fitness landscape is ‘step-like’ near the threshold (see Figure 2A). The time evolution of the probability distribution of stability in this mutational model with the flat ‘step-like’ landscape is given by [1]

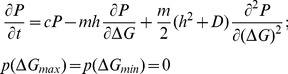
(2)where, 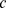 is a constant related to the birth rate of the population, 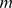 is the mutation rate per gene (or protein), 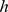 and 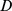 are the average and variance, respectively, of the distribution of stability changes upon mutation. Formally, 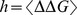 and 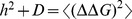, where 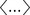 denotes the average over all possible mutations and 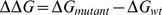. The second derivative in equation 2 describes diffusion, while drift is captured by the first derivative (in the right hand side of the equation). Using the long-time limit solution 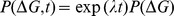 [1], we require the steady state solution to be the eigenfunction of the differential equation
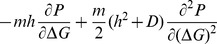
(3)subject to the boundary conditions. Thus, the steady state solution – within a normalization constant 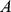 – is given by
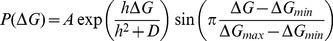
(4)


Noticing one-to-one relation between folding speed (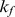) and barrier height (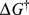), we employ similar idea to model the distribution of barrier height to ultimately predict the folding speed distribution. We use the same diffusion-drift model where mutations alter the free energy barrier of folding instead of folding stability. Analogous to the stability model, we impose two boundary conditions, 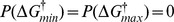, at the two extremities of the free energy barrier, 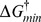 and 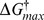 (see Figure 2B). On one hand it is simply impossible to make proteins that fold faster than the speed limit of folding, setting the lower limit of the barrier 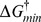. On the other hand, extremely slow folding proteins – if not folded at birth – even if highly stable will not be able to fold in time before degradation. Stated differently, for functional reasons, proteins would require to fold before their lifetime (inside the cell) expires. Also, slow folding proteins would be a potential hazard due to unfolded-state induced aggregation propensity. This sets a selection pressure against slow folding proteins with extremely high barriers (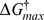). Similar to the stability model, we assume a flat-fitness landscape for 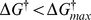, with a severe drop in fitness for 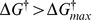 (Figure 2B). In reality, fitness can gradually decrease around the threshold value of 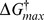. However, in order to keep the calculation simple and analogous to the work of Shakhnovich *et al.*, we make the simplifying assumption of a ‘step-like’ fitness function. Thus the model assumes all proteins are subjected to a single global constraint of lifetime implying a single value of 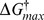. Noticing the exact analogy between the model for the stability and the barrier height, the predicted distribution for the free energy barrier can be easily obtained by replacing the stability (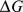) by the barrier height 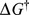 in equation 4. Thus,
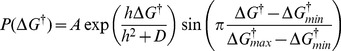
(5)where, 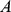 is a normalization constant, 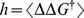, 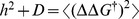; 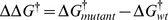, and 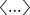 denotes the average over all possible mutations of barrier height. Three parameters of the model 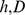, and 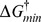, can be estimated from the literature. From the dataset of 858 mutations across 24 different proteins [30], we find 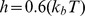 and 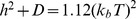; 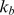 is the Boltzmann constant and 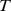 is the room temperature.

The lower limit of the barrier is assumed to be zero, (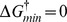), consistent with barrier-less folding proteins that define the speed limit of folding [25], [26].

Now we focus on the determination of 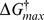. We hypothesize the lower speed limit i.e. the maximum folding time (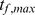) – setting the upper limit of folding barrier (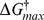) – has to be less than the protein halflife (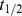). Experimentally reported halflife measures the time scale over which the copy number of a given protein, upon inhibition of synthesis, decreases by half [27]. This timescale does not distinguish between unfolded or folded state degradation, instead simply provides an estimate of the lifetime of a protein inside a cell. Based on this definition of halflife, it is natural to expect that proteins would be required to fold in a timescale lower than their halflife. Assuming lifetime distribution to be Poisson, average lifetime (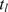) and halflife (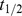) are related 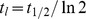. If the average folding time of a given protein is 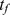, the probability of *folding before degradation* (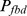) is
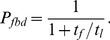
(6)


Clearly, if 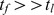 most of the proteins will be degraded before folding. At the other extreme if 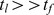, almost all of the proteins will be folded before degradation. It is also important to note, even if 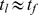, nearly 50% of the proteins will be degraded before folding which is not very efficient either. Thus we do not assume the boundary condition due to the maximum folding time to be exactly equal to the average lifetime of the fastest degrading protein. Instead, we fit topology-based folding speed distribution to determine the maximum allowed folding time for the diffusion-drift model. We find the best fit value of 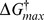 to be 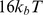, yielding the maximum folding time 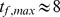 seconds (for Yeast distribution). In the above we used the speed-barrier height relation 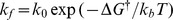 and 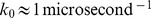. The numerical value of 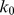 is consistent with several estimates of folding speed limit [21], [25], [26], [31], [32].

Figure 1 shows the best fit distribution is in reasonable agreement with the Yeast distribution. The implication of this is threefold: i) the diffusion-drift model provides an independent test of our topology-based model prediction for the distribution of folding kinetics; ii) 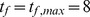 seconds and 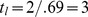 min (for the fastest degrading protein in Yeast) argues even the fastest degrading protein in Yeast has roughly 96% probability of folding before the expiration of its lifetime. This supports the hypothesis that the slowest folding processes may be constrained by protein lifetime allowing sufficient chance for proteins to fold before degradation; iii) the assumption of flat-fitness landscape is reasonable. This implies proteome folding kinetics is not subjected to any major selection criteria to make it faster other than the primary requirement of staying sufficiently below the maximum allowed timescale set by protein halflife. However, it can not be ruled out that there are other secondary pressures to alter folding kinetics that can further improve the agreement between the diffusion-drift and topology-based model of folding kinetics. We have also fitted E.coli speed distribution with the diffusion-drift model, yielding 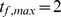 seconds (data not shown). However we do not provide details since a corresponding comparison with lifetime is not possible due to lack of lifetime information for E.coli proteome.

Diffusion-drift mutation model makes further prediction on the upper limit of the number of mutations per portion of the genome encoding essential genes per replication. As mentioned above, long time limit solution is given by 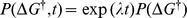. In order for the population to survive, we require 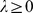. This requirement sets an upper limit on the number of mutations per portion of the genome encoding essential genes per replication. This limit can be obtained in terms of 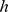, 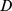, 
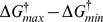
 (see equation 8 from [1] for details). Using the values for the parameters noted above, our estimate for the upper limit is 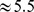. This is indeed close to 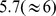 predicted by Shakhnovich *et al.* from the consideration of the stability distribution and matches well with experiments [1].

### Proteome folding time is lower than the lifetime

**Figure 3:**
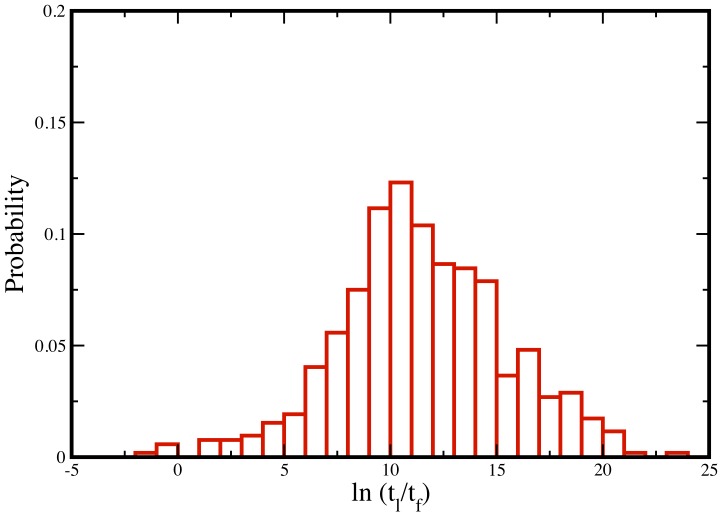
Distribution of the ratio of protein lifetime and protein folding time.

The analysis above provides indirect support to the hypothesis that proteome lifetime may limit folding kinetics. We further test this hypothesis by directly plotting the distribution of average lifetime (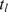 converted from experimentally measured halflife) values [27] for Yeast proteome (Figure 1 in green). It is evident that the folding time and lifetime distributions are well separated. However, we also notice slight overlap between the two time scales at the boundary. This observation, at first, may indicate existence of some proteins for which the folding time may be higher than the lifetime, implying a possible contradiction to our hypothesis that protein folding is faster than degradation. In order to further test the validity of our hypothesis, we directly compare these measured lifetime values [27] and predicted folding times for each individual proteins. We select proteins from our list – used to predict the folding time in the Yeast proteome – for which lifetimes are known [27]. We compute the ratio of the lifetime and folding time for each protein in our dataset (Table S3). Figure 3 shows the distribution of the ratios of these two time scales. We find less than 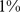 of the proteome (4 out of 520 proteins in our list) has a folding time higher than their lifetime. The overwhelming number of proteins with a lower folding time than their lifetime, further supports the hypothesis that the lower limit of protein folding speed is indeed bounded by protein lifetime.

Although 1% is a minor fraction, one can further reason these possible exceptions. First, chaperones can play an important role to facilitate folding [5], [33]–[35]. Chaperones can favorably alter the ratio of lifetime and folding time to help proteins escape the selection against degradation. Second, it is possible that the kinetics of the slowest folding domains are altered due to possible interdependence between multiple domains [36], an aspect not included in our model. Third, it should also be noted that the reported halflife in the work of O′Shea *et al.* [27] has an inherent uncertainty of a factor of two. In order to determine if any of the reasons mentioned above may be responsible, we further studied in detail the four proteins (corresponding open reading frames of YER070W, YFL041W, YJL200C and YLR304C) for which the predicted folding time is higher than the lifetime. We find three of these proteins (YFL041W, YJL200C and YLR304C) have folding time within twice their average lifetime, within the measurement uncertainty [27]. The only protein that has significantly higher folding time (fourfold higher than the lifetime) is YER070W with 80% probability of degradation before folding. However, it is interesting to note that this protein is also one of the highly abundant (top 5%) protein in the Yeast proteome [23]. The high abundance is likely due to its important biological function of facilitating synthesis of DNA. Furthermore, high abundance may offset the effect of slow folding ensuring enough copies (in absolute numbers) of the protein are present inside the cell despite the low probability of folding before degradation. Moreover, this protein has eighteen chaperone interaction partners as reported in ChaperoneDB database [28]. While the exact role of such unusually high number of chaperones to folding speed is not known at this time, it may be possible that some specific chaperones from this list or the entire chaperone network – in concert – facilitate folding of this protein in reasonable time scale to lower the burden of degradation.

## Conclusions

In summary, we predict the folding time distributions for E.coli and Yeast proteome weighted by protein expression levels. We make four key observations. First, we notice E.coli and Yeast have broad distributions of folding speed with roughly similar features and ranges of the distribution. Second, the underlying distribution is reasonably explained by an independent model of diffusion-drift of mutations in free energy barrier on a “flat-fitness landscape” with two boundary conditions. While the boundary at the upper speed limit (minimum folding time) is determined by barrierless folding proteins, we find the maximum folding time to be 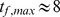 seconds (for Yeast proteome). Comparing this with the average lifetime of the fastest degrading protein (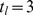 min), we find even the fastest degrading protein in Yeast has roughly 96% probability of folding before the expiration of its lifetime. This supports the hypothesis that the slowest folding time may be bounded by protein lifetime allowing sufficient chance for proteins to fold before degradation. Third, direct comparison between measured lifetime and predicted folding time shows 99% of the proteome has a folding time less than the corresponding lifetime. Finally, the reasonable agreement between the topology-based speed distribution and the diffusion-drift model on “flat-fitness landscape” further justifies the assumption of flat-fitness landscape. This implies the primary selection pressure for proteome folding kinetics is perhaps to outrun degradation only.

## Supporting Information

Table S1
**Dataset of folding time and abundance for E.coli proteome.** First column reports protein name as reported in O′Brien *et al.* [24]; second column reports 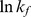 where 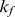 is the folding speed (in the units of 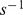) for the slowest folding domain; third column reports abundance value (in ppm) from PaxDB Integrated list [23].(PDF)

Table S2
**Dataset of folding time and abundance for Yeast proteome.** First column reports Open Reading Frame as reported in YRC [22]; second column reports 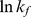 where 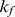 is the folding speed for the slowest folding domain in the units of 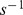; third column reports abundance value (in ppm) from PaxDB Integrated list [23].(PDF)

Table S3
**Dataset of folding time and halflife for Yeast proteome.** First column reports Open Reading Frame as reported in YRC [22]; second column reports halflife (in minutes) from O′Shea *et al.* [27]; third column reports 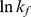 where 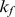 is the folding speed for the slowest folding domain in the units of 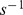.(PDF)
