# Supplementary material for: Proteome Folding Kinetics Is Limited by Protein Halflife
Source: PLoS One. 2014 Nov 13;9(11):e112701. doi: 10.1371/journal.pone.0112701 (PMC4231061; doi:10.1371/journal.pone.0112701)
Supplement: Table S1 — Dataset of folding time and abundance for E.coli proteome. First column reports protein name as reported in O′Brien et al. [24]; second column reports where is the folding speed (in the units of ) for the slowest folding domain; third column reports abundance value (in ppm) from PaxDB Integrated list [23]. (PDF) [file pone.0112701.s001.pdf]

| Protein Name | $lnk_f$ | Abundance |
|---|---|---|
| 3MG2_ECOLI | 4.6692 | 1.98506 |
| 6PGD_ECOLI | 0.7288 | 2232.37 |
| 6PGL_ECOLI | -1.8233 | 539.735 |
| AAT_ECOLI | 1.0725 | 1643.42 |
| ABDH_ECOLI | -0.3345 | 0.941557 |
| ACCC_ECOLI | -3.4208 | 397.754 |
| ACON2_ECOLI | 0.2416 | 1251.93 |
| ACP_ECOLI | 8.728 | 6378.67 |
| ACRA_ECOLI | 2.7442 | 229.931 |
| ACRB_ECOLI | 3.4081 | 18.5395 |
| ACRR_ECOLI | 9.3158 | 1.14548 |
| ACYP_ECOLI | 3.005 | 21.6036 |
| ADA_ECOLI | 5.3504 | 0.295255 |
| ADIA_ECOLI | -0.1103 | 6.45736 |
| ADPP_ECOLI | 0.6182 | 44.4978 |
| AEEP_ECOLI | 0.9357 | 10.1902 |
| AGAS_ECOLI | 2.4445 | 0.234426 |
| AHPF_ECOLI | 1.2523 | 448.903 |
| AIDB_ECOLI | -0.6159 | 32.9051 |
| AK3_ECOLI | -2.1097 | 479.45 |
| ALDA_ECOLI | -0.7045 | 606.825 |
| ALKB_ECOLI | -2.4938 | 3.49717 |
| ALKH_ECOLI | 0.6931 | 1204.25 |
| ALLA_ECOLI | 5.656 | 0.36692 |
| ALLB_ECOLI | 4.4275 | 0.678715 |
| ALLC_ECOLI | 0.6644 | 1.4679 |
| ALR1_ECOLI | 0.8831 | 35.8442 |
| ALSB_ECOLI | 2.5311 | 11.6968 |
| ALSE_ECOLI | 1.2644 | 1.10913 |
| ALSK_ECOLI | 3.3397 | 2.18932 |
| AMID_ECOLI | 1.5296 | 2.72275 |
| AMO_ECOLI | -7.3757 | 0.669372 |
| AMPA_ECOLI | -6.6634 | 286.448 |
| AMPC_ECOLI | -0.9461 | 1.87755 |
| AMPM_ECOLI | -3.4169 | 388.231 |
| AMPN_ECOLI | -3.7257 | 365.41 |
| AMPP_ECOLI | -1.713 | 330.962 |
| AMTB_ECOLI | -3.6597 | 194.722 |
| APBE_ECOLI | -2.269 | 1.1254 |
| APHA_ECOLI | 0.8545 | 18.2579 |
| APT_ECOLI | 2.4415 | 362.998 |
| ARAA_ECOLI | -0.2009 | 2.64295 |
| ARAF_ECOLI | 2.9739 | 1.45842 |
| ARCA_ECOLI | 5.3605 | 959.809 |
| ARCB_ECOLI | 8.1534 | 67.5413 |
| ARGB_ECOLI | -1.949 | 178.519 |
| ARGR_ECOLI | 7.5397 | 28.3436 |
| ARLY_ECOLI | 2.6375 | 293.796 |
| ARNA_ECOLI | -2.6435 | 60.6974 |
| AROA_ECOLI | -2.7138 | 219.141 |
| AROE_ECOLI | 2.2709 | 15.3119 |
| AROG_ECOLI | -1.7652 | 1017.26 |
| AROK_ECOLI | 1.5904 | 155.148 |
| ASCG_ECOLI | 3.6605 | 1.01411 |
| ASNC_ECOLI | 4.3507 | 106.665 |
| ASPA_ECOLI | 1.5817 | 847.518 |
| ASPG1_ECOLI | -1.7825 | 40.2925 |
| ASPG2_ECOLI | -2.2635 | 94.4783 |
| ASSY_ECOLI | 1.5896 | 1315.1 |
| ASTB_ECOLI | 0.6293 | 5.76732 |

| | | |
|---|---|---|
| ATKB_ECOLI | 1.998 | 2.18265 |
| ATPD_ECOLI | 6.8153 | 795.891 |
| ATPE_ECOLI | 1.7461 | 822.414 |
| ATPF_ECOLI | 11.1251 | 1009.81 |
| ATPG_ECOLI | 2.4335 | 762.651 |
| ATPL_ECOLI | 7.8523 | 393.284 |
| ATZN_ECOLI | 4.3647 | 30.158 |
| AZOR_ECOLI | 0.958 | 97.582 |
| BARA_ECOLI | 7.3523 | 3.65393 |
| BCCP_ECOLI | 3.5581 | 657.147 |
| BFR_ECOLI | 5.7408 | 333.098 |
| BGAL_ECOLI | -6.2437 | 0.397935 |
| BIOA_ECOLI | 2.2027 | 18.2616 |
| BIOF_ECOLI | 1.6431 | 8.40863 |
| BIOH_ECOLI | -0.9994 | 23.5402 |
| BIRA_ECOLI | 0.5258 | 18.0347 |
| BLC_ECOLI | 0.365 | 15.5287 |
| BOLA_ECOLI | 7.1884 | 207.136 |
| BTUB_ECOLI | -5.7029 | 15.6533 |
| BTUD_ECOLI | 1.5693 | 0.678346 |
| BTUF_ECOLI | 4.0079 | 0.438125 |
| CAIB_ECOLI | -0.653 | 0.410841 |
| CAN_ECOLI | 3.9531 | 522.967 |
| CAPP_ECOLI | -3.2628 | 1177.8 |
| CARA_ECOLI | -1.4245 | 1205.34 |
| CARB_ECOLI | -0.6536 | 912.706 |
| CATE_ECOLI | -6.1959 | 202.175 |
| CBL_ECOLI | 4.1976 | 0.970135 |
| CBPA_ECOLI | 8.3407 | 284.857 |
| CCME_ECOLI | 1.6302 | 0.564654 |
| CDD_ECOLI | 3.107 | 96.0709 |
| CDH_ECOLI | -0.398 | 6.69197 |
| CEDA_ECOLI | 7.8588 | 9.94073 |
| CH10_ECOLI | 1.4988 | 7208.12 |
| CH60_ECOLI | 1.6198 | 10660.6 |
| CHAB_ECOLI | 7.6974 | 26.1299 |
| CHEA_ECOLI | 4.8862 | 268.165 |
| CHEW_ECOLI | 0.8404 | 158.804 |
| CHEY_ECOLI | 5.4002 | 297.556 |
| CHEZ_ECOLI | 9.2932 | 531.622 |
| CIRA_ECOLI | 2.1272 | 881.121 |
| CISY_ECOLI | 6.0497 | 1232.26 |
| CLCA_ECOLI | -4.6151 | 1.58532 |
| CLPA_ECOLI | 4.2252 | 281.925 |
| CLPB_ECOLI | 1.0327 | 1570.08 |
| CLPP_ECOLI | 2.2169 | 1706.92 |
| CLPS_ECOLI | 4.4857 | 10.8579 |
| CNU_ECOLI | 10.7096 | 57.956 |
| COAA_ECOLI | -0.8514 | 19.6279 |
| COABC_ECOLI | -1.7314 | 62.2139 |
| COAE_ECOLI | 2.3107 | 1.69349 |
| CODA_ECOLI | -1.2225 | 431.291 |
| CRA_ECOLI | -0.5529 | 46.1257 |
| CRP_ECOLI | 3.5844 | 773.158 |
| CSPA_ECOLI | 2.6691 | 1747.92 |
| CSRA_ECOLI | 8.3981 | 774.198 |
| CUEO_ECOLI | -2.3677 | 24.1389 |
| CUER_ECOLI | 10.0135 | 5.57179 |
| CUTA_ECOLI | 3.3985 | 124.208 |
| CYAY_ECOLI | 9.1152 | 62.2048 |

| | | |
|---|---|---|
| CYNR_ECOLI | 5.1379 | 3.67354 |
| CYNS_ECOLI | 6.683 | 1.31994 |
| CYSJ_ECOLI | 2.3504 | 395.855 |
| CYSM_ECOLI | 2.6197 | 183.311 |
| DACA_ECOLI | 0.1635 | 126.707 |
| DACB_ECOLI | 8.8633 | 2.23862 |
| DACC_ECOLI | 0.3027 | 117.72 |
| DAPA_ECOLI | 0.4187 | 570.497 |
| DAPB_ECOLI | 3.4986 | 233.643 |
| DAPD_ECOLI | 0.8817 | 2148.41 |
| DCD_ECOLI | -2.356 | 137.731 |
| DCEA_ECOLI | -1.6489 | 1681.22 |
| DCEB_ECOLI | -2.329 | 1010.56 |
| DEF_ECOLI | 2.237 | 265.728 |
| DEGP_ECOLI | 0.4791 | 567.919 |
| DEGS_ECOLI | 0.9762 | 0.835132 |
| DEOC_ECOLI | 1.0878 | 457.638 |
| DEOD_ECOLI | -1.7564 | 937.471 |
| DGAL_ECOLI | 2.9161 | 960.428 |
| DHAK_ECOLI | 1.7196 | 137.062 |
| DHAS_ECOLI | -0.0564 | 1791.96 |
| DHSA_ECOLI | 1.9722 | 394.336 |
| DHSB_ECOLI | 2.1924 | 357.481 |
| DHSC_ECOLI | 8.5467 | 293.638 |
| DHSD_ECOLI | 10.0083 | 362.902 |
| DIAA_ECOLI | 3.4857 | 53.2721 |
| DINI_ECOLI | 5.797 | 7.55526 |
| DKGA_ECOLI | -0.298 | 249.993 |
| DKSA_ECOLI | 10.0167 | 741.209 |
| DLGD_ECOLI | 3.7672 | 0.850287 |
| DNAA_ECOLI | 6.9647 | 33.0282 |
| DNAB_ECOLI | 5.9226 | 74.9908 |
| DNAJ_ECOLI | 4.5602 | 263.487 |
| DNAK_ECOLI | 7.0606 | 8986.85 |
| DNLJ_ECOLI | -0.0241 | 41.0853 |
| DOSP_ECOLI | 3.7044 | 0.299548 |
| DPO1_ECOLI | 1.4558 | 258.202 |
| DPO2_ECOLI | 3.4123 | 1.30201 |
| DPO3A_ECOLI | -0.7125 | 9.47241 |
| DPO3B_ECOLI | 1.5028 | 100.803 |
| DPO3X_ECOLI | 2.8168 | 16.7952 |
| DPO4_ECOLI | 3.3179 | 0.976523 |
| DPS_ECOLI | 6.7958 | 1301.26 |
| DSBA_ECOLI | 3.2112 | 115.479 |
| DSBB_ECOLI | 7.7564 | 49.4535 |
| DSBC_ECOLI | 3.4499 | 387.723 |
| DSBD_ECOLI | 0.8726 | 0.354836 |
| DSBG_ECOLI | 3.1301 | 15.6797 |
| DUT_ECOLI | 1.6399 | 391.496 |
| DXR_ECOLI | 2.5523 | 0.83321 |
| DXS_ECOLI | 2.7834 | 72.0779 |
| DYR_ECOLI | 0.9045 | 70.1841 |
| ECOT_ECOLI | 7.8221 | 19.2802 |
| EFTS_ECOLI | 4.1831 | 4006.84 |
| EFTU1_ECOLI | 0.9841 | 13313.2 |
| EFTU2_ECOLI | 1.068 | 15497.5 |
| ELBB_ECOLI | -0.1587 | 434.39 |
| END4_ECOLI | -1.6694 | 28.9956 |
| ENO_ECOLI | -1.4465 | 11218. |
| ENTB_ECOLI | -0.1826 | 720.197 |

| | | |
|---|---|---|
| ENTC_ECOLI | 0.7956 | 287.014 |
| ENTF_ECOLI | 0.902 | 301.979 |
| ENTH_ECOLI | 0.8994 | 148.33 |
| ENVZ_ECOLI | 1.5958 | 0.816039 |
| ERA_ECOLI | 0.0353 | 44.4481 |
| EUTB_ECOLI | -0.8283 | 10.6671 |
| EUTD_ECOLI | 2.7249 | 2.48576 |
| EUTL_ECOLI | 1.3142 | 35.1317 |
| EUTS_ECOLI | 2.2253 | 0.946712 |
| EVGA_ECOLI | 4.2671 | 67.7984 |
| EX1_ECOLI | 2.7422 | 38.6206 |
| EX3_ECOLI | -1.8703 | 130.732 |
| EXBD_ECOLI | 6.6234 | 261.489 |
| F16PA_ECOLI | 0.8085 | 323.79 |
| FABB_ECOLI | 0.5448 | 1776.8 |
| FABD_ECOLI | 4.0261 | 1087.98 |
| FABF_ECOLI | 0.4195 | 381.345 |
| FABG_ECOLI | 0.0345 | 828.349 |
| FABH_ECOLI | 1.5334 | 821.25 |
| FABI_ECOLI | -0.9418 | 1679.76 |
| FADH_ECOLI | -3.0208 | 1.66946 |
| FADL_ECOLI | 2.8765 | 10.087 |
| FADR_ECOLI | 7.4759 | 96.9849 |
| FCTA_ECOLI | -0.4019 | 0.390677 |
| FDHF_ECOLI | 1.0341 | 0.140493 |
| FDNG_ECOLI | 0.7384 | 0.518928 |
| FDNH_ECOLI | 6.6875 | 6.08655 |
| FECA_ECOLI | -7.5908 | 14.4835 |
| FENR_ECOLI | 1.9892 | 52.3378 |
| FEOB_ECOLI | 2.0782 | 306.893 |
| FEOC_ECOLI | 6.2772 | 18.9072 |
| FEPE_ECOLI | 4.9707 | 19.7422 |
| FETP_ECOLI | 9.1484 | 814.922 |
| FHUA_ECOLI | -8.8954 | 99.3106 |
| FIMC_ECOLI | 0.2627 | 2.37696 |
| FIMD_ECOLI | 4.6737 | 0.383725 |
| FIS_ECOLI | 10.3215 | 1060.94 |
| FKBA_ECOLI | 1.2244 | 746.362 |
| FLAV_ECOLI | 1.8743 | 1482.26 |
| FLHC_ECOLI | 8.1394 | 1.86673 |
| FLHD_ECOLI | 8.8838 | 8.17424 |
| FOCA_ECOLI | 1.4166 | 114.656 |
| FOLB_ECOLI | 2.1313 | 54.0134 |
| FOLC_ECOLI | -1.0599 | 58.5888 |
| FRE_ECOLI | 2.198 | 91.56 |
| FSAA_ECOLI | 2.6234 | 19.0966 |
| FTSH_ECOLI | 0.3642 | 455.626 |
| FTSK_ECOLI | 7.3722 | 39.6423 |
| FTSN_ECOLI | 4.0937 | 28.7453 |
| FTSP_ECOLI | -0.0083 | 1.42913 |
| FTSQ_ECOLI | 5.5348 | 5.40363 |
| FTSY_ECOLI | 0.9716 | 190.486 |
| FUCI_ECOLI | -1.7162 | 3.59103 |
| FUCM_ECOLI | 7.7952 | 84.9926 |
| FUMC_ECOLI | 2.2012 | 80.779 |
| FUR_ECOLI | 8.4281 | 575.381 |
| G3P1_ECOLI | -1.1262 | 12772.5 |
| GABD_ECOLI | -0.9123 | 102.816 |
| GABT_ECOLI | 1.8581 | 67.4121 |
| GALE_ECOLI | 1.649 | 104.015 |

| | | |
|---|---|---|
| GALU_ECOLI | -3.6028 | 386.025 |
| GATZ_ECOLI | 8.8331 | 2190.98 |
| GCL_ECOLI | -1.5025 | 1.17101 |
| GCSH_ECOLI | 1.9459 | 1520.45 |
| GCST_ECOLI | 2.5258 | 428.538 |
| GFCB_ECOLI | 1.5944 | 0.412504 |
| GGT_ECOLI | 2.1478 | 15.517 |
| GLF_ECOLI | 8.0907 | 376.427 |
| GLGA_ECOLI | 1.8506 | 300.909 |
| GLGS_ECOLI | 9.7351 | 26.0246 |
| GLK_ECOLI | 1.1694 | 76.8464 |
| GLMS_ECOLI | -3.6068 | 340.63 |
| GLMU_ECOLI | -3.3163 | 89.9861 |
| GLNE_ECOLI | 4.6717 | 18.8469 |
| GLNH_ECOLI | 3.9854 | 1040.36 |
| GLNK_ECOLI | 3.7814 | 234.528 |
| GLPD_ECOLI | -5.5931 | 754.539 |
| GLPE_ECOLI | 2.4391 | 35.6372 |
| GLPG_ECOLI | 5.073 | 0.553535 |
| GLPK_ECOLI | -2.124 | 835.023 |
| GLPQ_ECOLI | -1.3465 | 426.915 |
| GLPX_ECOLI | 1.3078 | 7.23871 |
| GLRX1_ECOLI | 5.5338 | 99.5319 |
| GLRX2_ECOLI | 8.3202 | 474.111 |
| GLRX3_ECOLI | 5.7273 | 521.463 |
| GLRX4_ECOLI | 4.1516 | 1401.05 |
| GLSA1_ECOLI | 0.8449 | 83.9945 |
| GLUQ_ECOLI | -0.3224 | 2.24088 |
| GLYA_ECOLI | 1.1769 | 7872.73 |
| GM4D_ECOLI | 7.2757 | 108.282 |
| GMHA_ECOLI | 3.0907 | 802.798 |
| GMHB_ECOLI | 0.255 | 53.1539 |
| GNTK_ECOLI | 1.9368 | 7.72387 |
| GPMA_ECOLI | 0.9319 | 4968.94 |
| GREB_ECOLI | 3.8302 | 10.8837 |
| GRPE_ECOLI | 2.5398 | 1431.48 |
| GSH1_ECOLI | -3.5165 | 131.68 |
| GSHR_ECOLI | 2.1616 | 105.666 |
| GSIB_ECOLI | -6.3974 | 35.128 |
| GSP_ECOLI | -1.1759 | 121.896 |
| GSTA_ECOLI | 6.0306 | 238.562 |
| GUDD_ECOLI | 0.2588 | 1.83074 |
| GYRA_ECOLI | 2.8751 | 412.659 |
| GYRB_ECOLI | 0.3125 | 430.993 |
| HCHA_ECOLI | -4.3522 | 404.053 |
| HDEA_ECOLI | 7.7975 | 5469.68 |
| HEM3_ECOLI | 3.9624 | 3.70543 |
| HEMN_ECOLI | -0.9677 | 13.7252 |
| HFLD_ECOLI | 3.7588 | 31.8593 |
| HFQ_ECOLI | 5.9118 | 2239.93 |
| HHA_ECOLI | 9.8196 | 1.53668 |
| HIPA_ECOLI | 4.8875 | 6.03815 |
| HIPB_ECOLI | 9.5123 | 6.24735 |
| HIS7_ECOLI | 1.8084 | 140.958 |
| HIS8_ECOLI | 1.6487 | 397.924 |
| HISX_ECOLI | 1.8988 | 667.428 |
| HLDD_ECOLI | 2.3661 | 685.681 |
| HMP_ECOLI | 2.2697 | 15.5514 |
| HOLA_ECOLI | 4.9774 | 0.347018 |
| HOLB_ECOLI | 2.7245 | 11.5167 |

| | | |
|---|---|---|
| HOLC_ECOLI | 3.33 | 31.1164 |
| HOLD_ECOLI | 5.046 | 10.0948 |
| HOLE_ECOLI | 9.93 | 3.2284 |
| HPF_ECOLI | 6.3011 | 1195.86 |
| HPRT_ECOLI | -0.3149 | 254.056 |
| HSCA_ECOLI | 1.1724 | 41.2287 |
| HSCB_ECOLI | 9.9125 | 26.5723 |
| HSLJ_ECOLI | 2.1691 | 5.32876 |
| HSLO_ECOLI | 0.997 | 228.706 |
| HSLU_ECOLI | 5.5815 | 483.045 |
| HSLV_ECOLI | 3.4691 | 87.4801 |
| HTPG_ECOLI | 0.0955 | 701.251 |
| HYAE_ECOLI | 4.9674 | 3.82708 |
| HYBD_ECOLI | -0.486 | 12.9569 |
| HYCI_ECOLI | -0.5957 | 11.2153 |
| HYPC_ECOLI | 5.9138 | 2.22997 |
| HYPE_ECOLI | 2.0742 | 0.304402 |
| HYPF_ECOLI | 4.0298 | 0.607079 |
| IAAA_ECOLI | 6.7851 | 7.58611 |
| IADA_ECOLI | -0.0846 | 50.8797 |
| IDH_ECOLI | -1.2355 | 4673.77 |
| IF1_ECOLI | 3.2834 | 1300.9 |
| IF3_ECOLI | 3.8 | 2222.49 |
| IHFA_ECOLI | 7.7895 | 1928.3 |
| IHFB_ECOLI | 7.6625 | 1711.09 |
| ILVA_ECOLI | 1.31 | 18.4725 |
| ILVC_ECOLI | -0.1748 | 4181.44 |
| ILVH_ECOLI | 4.307 | 568.736 |
| INTS_ECOLI | 7.9405 | 3.77682 |
| IPYR_ECOLI | -1.6402 | 2273.57 |
| ISCA_ECOLI | 4.0246 | 13.6618 |
| ISCS_ECOLI | 1.189 | 1578.02 |
| ISCX_ECOLI | 7.3978 | 181.759 |
| ISPD_ECOLI | -1.9923 | 4.92721 |
| ISPE_ECOLI | 0.284 | 9.97063 |
| ISPF_ECOLI | 0.4059 | 116.023 |
| ISPH_ECOLI | 4.6288 | 155.897 |
| IVY_ECOLI | 5.4925 | 702.661 |
| K6PF1_ECOLI | 1.5275 | 999.878 |
| K6PF2_ECOLI | -0.7717 | 147.652 |
| KAD_ECOLI | 0.7915 | 4676.04 |
| KATG_ECOLI | 4.5795 | 526.086 |
| KBAY_ECOLI | -0.7718 | 1.33878 |
| KBL_ECOLI | 2.0717 | 241.892 |
| KCH_ECOLI | 4.2271 | 43.1643 |
| KCY_ECOLI | 1.7691 | 151.458 |
| KDGL_ECOLI | 11.284 | 10.1963 |
| KDGR_ECOLI | 2.4808 | 63.553 |
| KDPD_ECOLI | 7.8338 | 15.0033 |
| KDPE_ECOLI | 5.174 | 6.20337 |
| KDSB_ECOLI | -2.3922 | 97.2072 |
| KDSC_ECOLI | 0.6757 | 72.2214 |
| KDUI_ECOLI | 0.3278 | 19.013 |
| KGUA_ECOLI | 7.0147 | 143.014 |
| KPYK1_ECOLI | 1.7335 | 1228.64 |
| KTHY_ECOLI | 1.0106 | 37.1154 |
| LACI_ECOLI | 3.8924 | 14.6258 |
| LACY_ECOLI | 4.1803 | 0.428985 |
| LAMB_ECOLI | -6.7184 | 17.5456 |
| LEPA_ECOLI | 1.2361 | 224.396 |

| | | |
|---|---|---|
| LEP_ECOLI | 7.3976 | 92.7822 |
| LEXA_ECOLI | 1.6614 | 47.5841 |
| LFTR_ECOLI | 0.1996 | 3.4146 |
| LGUL_ECOLI | 2.739 | 279.059 |
| LIVK_ECOLI | 3.7014 | 540.988 |
| LOLA_ECOLI | 1.5211 | 108.471 |
| LOLB_ECOLI | 1.802 | 70.991 |
| LON_ECOLI | -1.7593 | 361.585 |
| LPLA_ECOLI | -1.619 | 39.4962 |
| LPP_ECOLI | 11.1335 | 1494.64 |
| LPTA_ECOLI | 2.2468 | 24.5895 |
| LPXA_ECOLI | 0.0704 | 213.91 |
| MAA_ECOLI | 0.8998 | 8.10879 |
| MACA_ECOLI | 3.3961 | 0.792844 |
| MALE_ECOLI | 4.56 | 447.791 |
| MALG_ECOLI | 4.0135 | 1.41761 |
| MALK_ECOLI | 0.6907 | 108.044 |
| MALY_ECOLI | 1.5149 | 14.7766 |
| MASY_ECOLI | 3.5748 | 809.95 |
| MASZ_ECOLI | 3.5635 | 135.04 |
| MAZG_ECOLI | 9.4105 | 7.62343 |
| MCP1_ECOLI | 8.1733 | 285.161 |
| MCP2_ECOLI | 8.0891 | 604.915 |
| MDAB_ECOLI | 1.1848 | 150.866 |
| MDH_ECOLI | 2.1613 | 9749.27 |
| MENC_ECOLI | 2.244 | 6.73512 |
| MEND_ECOLI | -0.8488 | 9.725 |
| MENF_ECOLI | -4.1822 | 0.412243 |
| METB_ECOLI | 1.2677 | 101.693 |
| METC_ECOLI | 0.4604 | 193.056 |
| METF_ECOLI | 0.814 | 238.731 |
| METH_ECOLI | 0.4534 | 36.9936 |
| METK_ECOLI | 4.8817 | 2620.05 |
| METN_ECOLI | 4.0511 | 51.9002 |
| MFD_ECOLI | -1.2528 | 47.2235 |
| MGSA_ECOLI | 2.3828 | 79.2304 |
| MHPC_ECOLI | -1.5559 | 4.90737 |
| MHPD_ECOLI | -2.9811 | 0.594781 |
| MIAA_ECOLI | 3.9931 | 6.15277 |
| MINE_ECOLI | 9.2823 | 840.612 |
| MIOC_ECOLI | 2.9077 | 240.578 |
| MLC_ECOLI | 2.8054 | 1.12063 |
| MLTA_ECOLI | 3.1655 | 4.09775 |
| MLTB_ECOLI | 5.5393 | 4.27845 |
| MNMC_ECOLI | -0.0932 | 12.9635 |
| MNME_ECOLI | 1.9048 | 188.792 |
| MNMG_ECOLI | 7.2733 | 31.9465 |
| MOAB_ECOLI | 0.5162 | 259.685 |
| MOAD_ECOLI | 3.4001 | 67.9474 |
| MOAE_ECOLI | 1.451 | 56.2452 |
| MOBA_ECOLI | 0.3326 | 7.37607 |
| MOBB_ECOLI | 8.4085 | 2.78126 |
| MODE_ECOLI | 3.8263 | 55.2143 |
| MOEA_ECOLI | 2.228 | 201.863 |
| MOEB_ECOLI | -0.379 | 5.66272 |
| MPGP_ECOLI | 6.0726 | 18.0895 |
| MQSA_ECOLI | 7.5861 | 1.07947 |
| MSRA_ECOLI | -1.1213 | 228.055 |
| MSRC_ECOLI | 2.912 | 154.647 |
| MTNN_ECOLI | -1.4215 | 414.552 |

| | | |
|---|---|---|
| MUKB_ECOLI | 3.2366 | 130.602 |
| MUKE_ECOLI | 7.5523 | 53.8522 |
| MUKF_ECOLI | 8.7495 | 13.4279 |
| MURA_ECOLI | -0.7496 | 281.548 |
| MURB_ECOLI | 2.0136 | 51.5076 |
| MURC_ECOLI | 0.3175 | 83.3788 |
| MURD_ECOLI | 0.5112 | 67.512 |
| MURE_ECOLI | 1.3949 | 121.344 |
| MURG_ECOLI | 1.8576 | 21.494 |
| MURI_ECOLI | 3.7668 | 17.587 |
| MUTH_ECOLI | -0.3041 | 1.12143 |
| MUTL_ECOLI | 0.2535 | 8.04081 |
| MUTS_ECOLI | -1.4423 | 10.308 |
| MUTT_ECOLI | 1.5306 | 3.83527 |
| MUTY_ECOLI | 5.3207 | 8.88006 |
| NADB_ECOLI | 2.174 | 8.79014 |
| NAGA_ECOLI | -0.7222 | 210.882 |
| NAGD_ECOLI | -0.4039 | 109.257 |
| NANA_ECOLI | 1.8035 | 29.3376 |
| NANK_ECOLI | 2.6422 | 0.610203 |
| NANM_ECOLI | 0.5223 | 1.65884 |
| NAPA_ECOLI | 1.4703 | 1.17382 |
| NAPD_ECOLI | 4.3165 | 1.03616 |
| NARG_ECOLI | 1.4637 | 57.5774 |
| NARH_ECOLI | 7.2793 | 43.8843 |
| NARI_ECOLI | 4.9703 | 3.45592 |
| NARL_ECOLI | 3.4944 | 356.96 |
| NARX_ECOLI | 8.1497 | 3.48861 |
| NCPP_ECOLI | 1.6164 | 23.306 |
| NDK_ECOLI | 2.3845 | 2529.65 |
| NFNB_ECOLI | 1.18 | 833.524 |
| NFSA_ECOLI | -0.1089 | 149.273 |
| NIKA_ECOLI | -5.5354 | 3.54541 |
| NIKR_ECOLI | 4.212 | 21.7702 |
| NIRD_ECOLI | 3.1578 | 0.569577 |
| NLPE_ECOLI | 2.0835 | 4.73141 |
| NLPI_ECOLI | 6.3086 | 9.58657 |
| NPD_ECOLI | -0.271 | 32.6847 |
| NRDH_ECOLI | 5.8527 | 71.9856 |
| NRFA_ECOLI | -0.3034 | 9.33768 |
| NRFB_ECOLI | 5.7911 | 1.99642 |
| NUDC_ECOLI | 2.2704 | 16.7055 |
| NUDE_ECOLI | 0.8048 | 72.9895 |
| NUDJ_ECOLI | 0.8524 | 7.21411 |
| NUDK_ECOLI | 0.5673 | 26.6264 |
| NUSA_ECOLI | 8.9368 | 1125.11 |
| NUSB_ECOLI | 7.1854 | 85.9805 |
| NUSG_ECOLI | 4.7441 | 682.053 |
| ODO1_ECOLI | -1.324 | 681.743 |
| ODO2_ECOLI | 1.8582 | 1086.19 |
| ODP1_ECOLI | -1.9647 | 2084.7 |
| ODP2_ECOLI | 2.9604 | 1848.15 |
| OMPA_ECOLI | -2.8582 | 6860.85 |
| OMPC_ECOLI | -5.0134 | 111.929 |
| OMPF_ECOLI | -3.8396 | 197.516 |
| OMPR_ECOLI | 6.7803 | 269.339 |
| OMPT_ECOLI | -5.7265 | 510.789 |
| OMPW_ECOLI | -3.5104 | 126.949 |
| OMPX_ECOLI | -2.1803 | 499.491 |
| OPGG_ECOLI | -9.4555 | 360.427 |

| | | |
|---|---|---|
| ORN_ECOLI | 2.9817 | 60.0414 |
| OSMC_ECOLI | 4.4629 | 1321.19 |
| OTC1_ECOLI | 1.5245 | 169.71 |
| OTSA_ECOLI | 0.9806 | 112.489 |
| OXC_ECOLI | -0.9409 | 2.99529 |
| OXYR_ECOLI | 5.7695 | 211.921 |
| PA1_ECOLI | -3.0586 | 14.141 |
| PAAC_ECOLI | 3.5521 | 4.11564 |
| PAAI_ECOLI | -0.1319 | 0.168561 |
| PABC_ECOLI | 0.0772 | 1.68032 |
| PAL_ECOLI | 3.0037 | 301.072 |
| PANB_ECOLI | -0.4115 | 726.898 |
| PANC_ECOLI | 2.2054 | 254.643 |
| PAND_ECOLI | 5.4772 | 240.8 |
| PANE_ECOLI | 0.0918 | 0.336476 |
| PARC_ECOLI | 10.4097 | 134.847 |
| PCKA_ECOLI | 1.778 | 628.28 |
| PDXA_ECOLI | -4.069 | 20.7349 |
| PDXH_ECOLI | 0.9039 | 195.035 |
| PDXJ_ECOLI | 0.797 | 245.53 |
| PDXK_ECOLI | 2.2641 | 99.8562 |
| PDXY_ECOLI | -0.2773 | 62.3459 |
| PEPT_ECOLI | 1.0457 | 128.99 |
| PFLA_ECOLI | 2.0875 | 76.9506 |
| PFLB_ECOLI | -11.2988 | 2621.54 |
| PGK_ECOLI | -0.5463 | 8342.74 |
| PHNP_ECOLI | -1.5188 | 5.36768 |
| PHOP_ECOLI | 5.0879 | 449.142 |
| PHOQ_ECOLI | 1.9908 | 14.0719 |
| PHR_ECOLI | 2.2133 | 3.75549 |
| PHSM_ECOLI | -2.9148 | 80.6575 |
| PMRD_ECOLI | 2.5593 | 6.2685 |
| PNP_ECOLI | 3.6074 | 1653.45 |
| PNTA_ECOLI | 1.4375 | 274.719 |
| PNTB_ECOLI | 0.1683 | 486.466 |
| POTD_ECOLI | 4.9589 | 327.303 |
| POTF_ECOLI | 4.3389 | 40.6008 |
| POXB_ECOLI | -0.5425 | 152.244 |
| PPA_ECOLI | 3.2856 | 7.76637 |
| PPB_ECOLI | 20.7662 | 1.97278 |
| PPIA_ECOLI | 4.0346 | 721.945 |
| PPIB_ECOLI | 2.3925 | 1711.23 |
| PPIC_ECOLI | 4.0961 | 155.85 |
| PPID_ECOLI | 3.7589 | 175.533 |
| PPK_ECOLI | 1.6171 | 39.5841 |
| PPTA_ECOLI | 7.1579 | 56.6336 |
| PPX_ECOLI | 1.6806 | 33.3303 |
| PRIA_ECOLI | 5.8182 | 0.169165 |
| PRIM_ECOLI | 3.9148 | 2.27272 |
| PROB_ECOLI | 1.4205 | 122.954 |
| PROX_ECOLI | 2.7924 | 245.705 |
| PRPR_ECOLI | 5.1717 | 5.96589 |
| PSPE_ECOLI | 2.8134 | 735.089 |
| PSPF_ECOLI | 1.7109 | 1.52451 |
| PSTS_ECOLI | 3.4218 | 218.542 |
| PT1_ECOLI | 6.8789 | 1228.04 |
| PTGA_ECOLI | 2.0492 | 4167.95 |
| PTGCB_ECOLI | 5.5084 | 437.579 |
| PTHP_ECOLI | 3.9462 | 2076.86 |
| PTM3C_ECOLI | 2.2238 | 145.076 |

| | | |
|---|---|---|
| PTNAB_ECOLI | 2.0998 | 804.77 |
| PTPB1_ECOLI | 1.4783 | 0.751216 |
| PTQA_ECOLI | 8.3967 | 5.39723 |
| PTSN_ECOLI | 2.3836 | 154.244 |
| PUR2_ECOLI | 2.5775 | 503.468 |
| PUR3_ECOLI | 1.412 | 76.4324 |
| PUR7_ECOLI | 3.7239 | 1930.08 |
| PUR8_ECOLI | 3.1917 | 728.691 |
| PURA_ECOLI | -0.2428 | 2503.45 |
| PURE_ECOLI | 3.3869 | 915.588 |
| PURK_ECOLI | 0.8516 | 64.6844 |
| PURR_ECOLI | 8.6081 | 259.091 |
| PURT_ECOLI | -1.965 | 528.916 |
| PUTA_ECOLI | -0.9858 | 43.8722 |
| PYRB_ECOLI | 2.2438 | 3595.11 |
| PYRC_ECOLI | -1.4455 | 1177.09 |
| PYRF_ECOLI | 0.9236 | 57.0971 |
| PYRG_ECOLI | 0.0938 | 638.611 |
| PYRH_ECOLI | -1.6454 | 523.182 |
| PYRI_ECOLI | 3.067 | 2026.53 |
| QOR2_ECOLI | 0.6162 | 13.9401 |
| RAIA_ECOLI | 6.1907 | 1032.22 |
| RBFA_ECOLI | 6.2371 | 583.031 |
| RBN_ECOLI | -3.018 | 7.56998 |
| RBSB_ECOLI | 3.2187 | 493.781 |
| RBSK_ECOLI | -3.0467 | 5.77977 |
| RCSC_ECOLI | 4.633 | 12.8925 |
| RCSD_ECOLI | 10.2066 | 23.4507 |
| RDGB_ECOLI | 2.0302 | 367.747 |
| RDGC_ECOLI | -1.3707 | 86.6124 |
| RECA_ECOLI | 9.6113 | 1375.88 |
| RECQ_ECOLI | 0.1671 | 1.92061 |
| RECX_ECOLI | 8.2792 | 3.28967 |
| RELB_ECOLI | 11.1465 | 40.9029 |
| RELE_ECOLI | 5.3226 | 39.369 |
| RF2_ECOLI | 7.279 | 570.864 |
| RF3_ECOLI | 0.2119 | 153.601 |
| RFAF_ECOLI | 2.8874 | 2.11518 |
| RFAG_ECOLI | -1.728 | 1.28204 |
| RHAA_ECOLI | -1.7081 | 0.585078 |
| RHAB_ECOLI | -1.7626 | 6.45994 |
| RHO_ECOLI | 3.2979 | 3340.96 |
| RIBA_ECOLI | 2.521 | 124.832 |
| RIBB_ECOLI | 5.083 | 298.746 |
| RIBD_ECOLI | 5.2865 | 50.9569 |
| RIDA_ECOLI | 1.9388 | 3910.67 |
| RIHA_ECOLI | -3.9089 | 138.896 |
| RIMN_ECOLI | 1.7197 | 39.8367 |
| RIR1_ECOLI | -8.0377 | 494.817 |
| RIR2_ECOLI | 3.8824 | 158.208 |
| RISA_ECOLI | 2.5434 | 281.53 |
| RL25_ECOLI | 2.6234 | 4246.44 |
| RL7_ECOLI | 5.0607 | 14543.5 |
| RLMA_ECOLI | -1.7251 | 5.56849 |
| RLMB_ECOLI | 0.5071 | 111.574 |
| RLMH_ECOLI | 1.3785 | 34.8046 |
| RLMI_ECOLI | 0.5396 | 27.9511 |
| RLUA_ECOLI | -0.1433 | 9.4589 |
| RLUC_ECOLI | 1.4556 | 24.8893 |
| RLUD_ECOLI | -0.7527 | 45.4299 |

| | | |
|---|---|---|
| RLUE_ECOLI | 1.2131 | 2.41944 |
| RLUF_ECOLI | 0.1131 | 8.49777 |
| RMLA2_ECOLI | -2.6574 | 21.8838 |
| RNB_ECOLI | -2.7377 | 231.127 |
| RND_ECOLI | 3.1229 | 8.92864 |
| RNE_ECOLI | 2.5226 | 273.504 |
| RNH_ECOLI | 0.4695 | 4.37517 |
| RNI_ECOLI | -1.2944 | 49.9805 |
| RNK_ECOLI | 2.5329 | 46.0841 |
| ROF_ECOLI | 4.8976 | 48.2277 |
| RPIA_ECOLI | 2.5897 | 1242.57 |
| RPIB_ECOLI | 2.0201 | 402.716 |
| RPOA_ECOLI | 4.8855 | 2981.46 |
| RPOC_ECOLI | 3.0926 | 3754.95 |
| RPOD_ECOLI | 8.581 | 310.202 |
| RPOE_ECOLI | 9.6687 | 53.01 |
| RPPH_ECOLI | 1.7417 | 39.756 |
| RRAA_ECOLI | 2.3101 | 375.931 |
| RRF_ECOLI | 5.8409 | 1764.72 |
| RRMF_ECOLI | 4.2912 | 173.629 |
| RS10_ECOLI | 4.0387 | 3472.67 |
| RS1_ECOLI | 2.3712 | 2649.13 |
| RS8_ECOLI | 4.7273 | 3828.28 |
| RSD_ECOLI | 9.147 | 20.6137 |
| RSEB_ECOLI | 2.6649 | 35.3171 |
| RSEP_ECOLI | 2.5191 | 3.52883 |
| RSMA_ECOLI | 0.7231 | 14.9838 |
| RSMC_ECOLI | 1.4432 | 35.0894 |
| RSMD_ECOLI | 1.9087 | 28.8863 |
| RSMF_ECOLI | 0.068 | 0.197008 |
| RSUA_ECOLI | 7.0976 | 139.836 |
| RTCA_ECOLI | -0.3893 | 0.44454 |
| RUTR_ECOLI | 8.0651 | 15.2391 |
| RUVA_ECOLI | 4.0453 | 79.8584 |
| RUVX_ECOLI | 2.1388 | 18.6398 |
| SDIA_ECOLI | 4.0009 | 12.8071 |
| SECB_ECOLI | 3.2046 | 2324.21 |
| SELB_ECOLI | 8.3237 | 27.8841 |
| SEQA_ECOLI | 8.0844 | 179.879 |
| SERA_ECOLI | 1.3318 | 2584.3 |
| SERC_ECOLI | 1.5232 | 1716.23 |
| SIXA_ECOLI | 1.1591 | 12.201 |
| SKP_ECOLI | 5.6544 | 2048.11 |
| SLT_ECOLI | 6.4404 | 13.4988 |
| SLYD_ECOLI | 5.352 | 1783.48 |
| SODC_ECOLI | -1.3407 | 96.6705 |
| SODF_ECOLI | 5.0989 | 1337.66 |
| SODM_ECOLI | 5.8897 | 1593.96 |
| SOXR_ECOLI | 9.5316 | 0.422779 |
| SPR_ECOLI | 3.5373 | 0.820977 |
| SRP54_ECOLI | 9.1352 | 124.678 |
| SSB_ECOLI | 1.1858 | 278.677 |
| SSPB_ECOLI | 4.1453 | 98.9388 |
| SUCC_ECOLI | 2.1122 | 2211.55 |
| SUCD_ECOLI | 0.3314 | 1950.06 |
| SUFA_ECOLI | 3.9197 | 60.0146 |
| SUFC_ECOLI | 4.9455 | 368.605 |
| SUFD_ECOLI | -4.6485 | 52.2038 |
| SUFE_ECOLI | 6.5845 | 3.72591 |
| SUFS_ECOLI | 0.1576 | 121.174 |

| | | |
|---|---|---|
| SUHB_ECOLI | 1.824 | 382.305 |
| SUPH_ECOLI | 5.0105 | 22.8818 |
| SURA_ECOLI | 3.8516 | 360.823 |
| SYA_ECOLI | -0.6924 | 626.077 |
| SYC_ECOLI | -1.1939 | 304.55 |
| SYDP_ECOLI | 3.4708 | 17.6227 |
| SYD_ECOLI | 0.2127 | 863.258 |
| SYH_ECOLI | 4.7201 | 432.677 |
| SYK1_ECOLI | -0.6477 | 765.3 |
| SYK2_ECOLI | -0.617 | 435.707 |
| SYL_ECOLI | 0.879 | 535.292 |
| SYM_ECOLI | 5.462 | 462.385 |
| SYQ_ECOLI | 3.3672 | 511.157 |
| SYT_ECOLI | -2.3264 | 1131.4 |
| SYY_ECOLI | 0.6199 | 534.711 |
| TADA_ECOLI | 3.6586 | 10.0433 |
| TALB_ECOLI | -0.6033 | 2457.39 |
| TAS_ECOLI | -1.0815 | 29.8486 |
| TATD_ECOLI | 0.6904 | 53.041 |
| TAUD_ECOLI | 20.2166 | 1.59533 |
| TDCF_ECOLI | 1.655 | 266.008 |
| TESA_ECOLI | 3.24 | 15.5247 |
| TESB_ECOLI | -0.8964 | 64.2727 |
| THIB_ECOLI | 4.589 | 6.44321 |
| THIF_ECOLI | -0.9471 | 36.7232 |
| THIO_ECOLI | 3.7636 | 1347.07 |
| THIS_ECOLI | 4.4575 | 19.1169 |
| THRC_ECOLI | 3.1843 | 1206.75 |
| THTM_ECOLI | -0.0129 | 256.273 |
| TIG_ECOLI | 3.3877 | 3826.41 |
| TILS_ECOLI | 2.734 | 7.43754 |
| TKT1_ECOLI | -1.051 | 1151.35 |
| TMCA_ECOLI | 1.7487 | 4.59833 |
| TNAA_ECOLI | 2.0735 | 2619.69 |
| TOLA_ECOLI | 5.3307 | 35.8598 |
| TOLB_ECOLI | -0.869 | 448.595 |
| TOLC_ECOLI | -3.1179 | 238.651 |
| TONB_ECOLI | 3.5001 | 72.5702 |
| TOP1_ECOLI | 3.4669 | 134.84 |
| TOP3_ECOLI | 2.9822 | 9.43328 |
| TORR_ECOLI | 5.122 | 1.97159 |
| TORS_ECOLI | 7.4738 | 4.02357 |
| TPIS_ECOLI | 0.5152 | 2582.1 |
| TPX_ECOLI | 2.0348 | 4678.16 |
| TREA_ECOLI | -1.8271 | 15.8814 |
| TRER_ECOLI | 4.4956 | 2.17531 |
| TRMA_ECOLI | 2.6464 | 42.1458 |
| TRMB_ECOLI | 1.7717 | 122.548 |
| TRMD_ECOLI | 1.094 | 19.0448 |
| TRPA_ECOLI | 0.3379 | 1659.72 |
| TRPB_ECOLI | 1.0371 | 1137.41 |
| TRPC_ECOLI | 0.3652 | 149.527 |
| TRPR_ECOLI | 10.6834 | 158.069 |
| TRUB_ECOLI | 4.3671 | 6.72581 |
| TRUD_ECOLI | -5.3619 | 40.8922 |
| TRXB_ECOLI | 1.5468 | 504.066 |
| TUSA_ECOLI | 2.9822 | 67.4759 |
| TUSB_ECOLI | 3.0239 | 4.05836 |
| TUSC_ECOLI | 2.1457 | 5.68647 |
| TUSD_ECOLI | 1.1339 | 8.08922 |

| | | |
|---|---|---|
| TUS_ECOLI | 4.3576 | 2.38202 |
| TYPH_ECOLI | 3.5002 | 63.3227 |
| TYRB_ECOLI | 1.5935 | 285.891 |
| TYRR_ECOLI | 4.7459 | 22.9026 |
| TYSY_ECOLI | 3.0299 | 230.974 |
| UBIC_ECOLI | 0.2828 | 7.15471 |
| UBID_ECOLI | 3.4149 | 58.1793 |
| UDP_ECOLI | -1.5062 | 2135.38 |
| ULAD_ECOLI | 1.8939 | 0.976689 |
| ULAE_ECOLI | 5.3247 | 0.758962 |
| UNG_ECOLI | 1.1127 | 35.214 |
| UPPS_ECOLI | 0.4008 | 33.62 |
| UPP_ECOLI | 0.0119 | 2298.75 |
| USHA_ECOLI | -1.2964 | 83.5377 |
| UVRB_ECOLI | 9.7575 | 36.1964 |
| UVRC_ECOLI | 9.6075 | 16.6248 |
| UVRD_ECOLI | 5.5274 | 100.2 |
| WECB_ECOLI | 2.6727 | 39.2572 |
| WRBA_ECOLI | -1.5158 | 1082.28 |
| XERD_ECOLI | 5.2917 | 2.10281 |
| XGPT_ECOLI | 1.5292 | 105.286 |
| XYLB_ECOLI | -2.3483 | 0.401687 |
| XYLS_ECOLI | -4.084 | 0.38201 |
| YACG_ECOLI | 8.1447 | 42.0497 |
| YAEQ_ECOLI | 1.0039 | 9.11726 |
| YAET_ECOLI | 3.569 | 165.583 |
| YAGE_ECOLI | 1.5567 | 41.3261 |
| YAHK_ECOLI | -0.7284 | 189.101 |
| YAJI_ECOLI | 0.9849 | 4.98114 |
| YAJL_ECOLI | 0.4384 | 68.7378 |
| YBAB_ECOLI | 7.8169 | 622.325 |
| YBAK_ECOLI | -0.3733 | 109.87 |
| YBAL_ECOLI | 3.7056 | 19.2712 |
| YBAQ_ECOLI | 8.9041 | 1.73636 |
| YBCJ_ECOLI | 4.6138 | 44.2745 |
| YBDL_ECOLI | -0.4076 | 0.396928 |
| YBED_ECOLI | 3.4968 | 1113.07 |
| YBFF_ECOLI | -0.5103 | 28.6609 |
| YBGC_ECOLI | 0.5998 | 40.8597 |
| YBGI_ECOLI | 3.2775 | 349.561 |
| YBGL_ECOLI | -0.2704 | 19.3173 |
| YBHB_ECOLI | -2.9285 | 47.0566 |
| YBHC_ECOLI | -3.6381 | 50.51 |
| YBIA_ECOLI | 4.3417 | 0.578514 |
| YBIC_ECOLI | 1.2931 | 346.847 |
| YCAC_ECOLI | 1.1911 | 93.2284 |
| YCDX_ECOLI | -0.1148 | 71.5636 |
| YCEB_ECOLI | 0.2661 | 28.1184 |
| YCEI_ECOLI | -0.1981 | 50.3851 |
| YCEM_ECOLI | 2.118 | 21.8269 |
| YCFH_ECOLI | 0.3882 | 36.5192 |
| YCGL_ECOLI | 4.3171 | 65.58 |
| YCHN_ECOLI | 2.0918 | 266.068 |
| YCIF_ECOLI | 8.5977 | 63.517 |
| YCIH_ECOLI | 5.0938 | 22.3964 |
| YCIK_ECOLI | -0.9098 | 22.8577 |
| YCIO_ECOLI | 1.7748 | 140.339 |
| YDCF_ECOLI | 1.5644 | 122.093 |
| YDFO_ECOLI | 4.2533 | 23.0558 |
| YDHF_ECOLI | 0.2091 | 31.9241 |

| | | |
|---|---|---|
| YDHR_ECOLI | 3.8368 | 595.114 |
| YDIB_ECOLI | 3.3402 | 1.26591 |
| YDIF_ECOLI | -2.9222 | 5.2584 |
| YDII_ECOLI | 0.4792 | 31.5089 |
| YDJA_ECOLI | 0.2834 | 539.515 |
| YDJH_ECOLI | -0.1044 | 0.316566 |
| YEAZ_ECOLI | 3.3357 | 25.7921 |
| YECD_ECOLI | -0.0685 | 110.198 |
| YEDF_ECOLI | 3.2126 | 118.222 |
| YEDY_ECOLI | -3.8322 | 1.09207 |
| YEFM_ECOLI | 9.0367 | 42.6043 |
| YEGP_ECOLI | 8.2258 | 237.454 |
| YEJL_ECOLI | 11.2052 | 202.416 |
| YFBM_ECOLI | 2.474 | 0.686064 |
| YFBU_ECOLI | 8.2539 | 454.223 |
| YFCD_ECOLI | 0.7505 | 100.691 |
| YFCE_ECOLI | -0.199 | 212.556 |
| YFCF_ECOLI | 8.7487 | 43.2842 |
| YFCG_ECOLI | 5.5988 | 3.39437 |
| YFEY_ECOLI | 0.2074 | 2.0049 |
| YFIH_ECOLI | -0.426 | 18.3875 |
| YFJZ_ECOLI | 3.5289 | 1.04196 |
| YGAV_ECOLI | 8.887 | 19.9934 |
| YGDK_ECOLI | 6.6433 | 14.5393 |
| YGFY_ECOLI | 9.9701 | 73.743 |
| YGFZ_ECOLI | 4.1646 | 532.381 |
| YGGS_ECOLI | 1.074 | 200.197 |
| YGHU_ECOLI | 4.3174 | 100.899 |
| YGIN_ECOLI | 4.5125 | 437.474 |
| YGJH_ECOLI | 1.6059 | 0.955842 |
| YGJK_ECOLI | -4.6743 | 0.33015 |
| YHAK_ECOLI | -5.9246 | 1.03584 |
| YHBO_ECOLI | 0.7791 | 7.57826 |
| YHBY_ECOLI | 5.3364 | 370.188 |
| YHCO_ECOLI | 5.4052 | 14.7551 |
| YHDH_ECOLI | -0.024 | 179.049 |
| YHFA_ECOLI | 3.7906 | 244.925 |
| YHHK_ECOLI | 5.4987 | 71.7462 |
| YHHW_ECOLI | -5.8044 | 72.7437 |
| YHHX_ECOLI | -1.0904 | 64.18 |
| YIAD_ECOLI | 3.1385 | 2.9451 |
| YIAJ_ECOLI | 2.6599 | 41.0376 |
| YIBA_ECOLI | 8.7236 | 6.60691 |
| YIDA_ECOLI | 3.3779 | 160.669 |
| YIGZ_ECOLI | 2.0774 | 5.84908 |
| YIHX_ECOLI | 7.6768 | 158.14 |
| YIIX_ECOLI | -1.16 | 1.07407 |
| YJBJ_ECOLI | 9.8245 | 1346.62 |
| YJBR_ECOLI | 6.4406 | 228.978 |
| YJHP_ECOLI | -0.6663 | 1.94084 |
| YJIA_ECOLI | 0.2024 | 48.1395 |
| YJJV_ECOLI | 0.9133 | 23.6574 |
| YLBA_ECOLI | -5.9746 | 40.2898 |
| YLII_ECOLI | 0.3067 | 0.278863 |
| YMDB_ECOLI | 0.6237 | 33.3527 |
| YNIC_ECOLI | 4.6262 | 93.3856 |
| YNJE_ECOLI | 0.3089 | 16.7404 |
| YQCC_ECOLI | 9.1032 | 7.43297 |
| YQFB_ECOLI | 1.93 | 158.62 |
| YQHD_ECOLI | 4.6642 | 140.358 |

| | | |
|---|---|---|
| YRBA_ECOLI | 6.3407 | 121.878 |
| YTFP_ECOLI | 1.0158 | 39.7828 |
| YTFQ_ECOLI | 2.7459 | 14.684 |
| ZAPB_ECOLI | 11.1471 | 2881.2 |
| ZINT_ECOLI | 0.7992 | 536.854 |
| ZIPA_ECOLI | 2.9097 | 34.2405 |
| ZNTR_ECOLI | 10.5423 | 16.1262 |
| ZNUA_ECOLI | 5.7443 | 1554.23 |
