# Supplementary material for: Proteome Folding Kinetics Is Limited by Protein Halflife
Source: PLoS One. 2014 Nov 13;9(11):e112701. doi: 10.1371/journal.pone.0112701 (PMC4231061; doi:10.1371/journal.pone.0112701)
Supplement: Table S2 — Dataset of folding time and abundance for Yeast proteome. First column reports Open Reading Frame as reported in YRC [22]; second column reports where is the folding speed for the slowest folding domain in the units of ; third column reports abundance value (in ppm) from PaxDB Integrated list [23]. (PDF) [file pone.0112701.s002.pdf]

Protein Name	lnk_f	Abundance
YAL005C	-1.008	8176.13
YAL012W	0.0827	1250.8
YAL036C	-0.8232	169.016
YAL054C	0.8474	12.7838
YAR007C	0.0176	88.8211
YAR015W	0.5259	458.126
YAR073W	4.0634	55.2365
YAR075W	10.73	36.8053
YBL002W	10.3077	2177.45
YBL003C	9.6808	1251.97
YBL007C	3.6146	159.776
YBL016W	5.5994	86.6626
YBL023C	1.5802	29.7486
YBL036C	0.8874	85.242
YBL041W	0.5798	52.7966
YBL045C	2.6272	302.069
YBL050W	8.3707	59.1412
YBL052C	6.6829	1.38574
YBL068W	0.6985	56.6699
YBL071W-A	5.4562	124.176
YBL075C	-0.4601	290.677
YBL078C	4.32	8.29383
YBL092W	7.0704	2504.49
YBL099W	-1.2054	859.364
YBL105C	4.5131	26.9852
YBL106C	2.7822	0.0747926
YBR006W	-0.9123	12.9192
YBR011C	-2.7881	1125.14
YBR014C	8.4328	5.19976
YBR019C	-7.2961	9.15151
YBR020W	0.3611	42.4474
YBR025C	8.6771	1274.36
YBR031W	4.0584	1903.46
YBR034C	-3.9063	152.469
YBR035C	1.0835	132.038
YBR082C	4.9411	1148.3
YBR087W	8.8626	24.0958
YBR088C	1.3361	153.092
YBR089C-A	8.0965	145.653
YBR097W	3.9846	0.833378
YBR109C	8.386	523.616
YBR117C	-1.914	6.52563
YBR121C	0.764	877.771
YBR143C	3.2399	679.185
YBR145W	-1.042	51.4057
YBR154C	4.3726	72.0715
YBR160W	4.2391	133.856
YBR164C	1.518	91.119
YBR169C	-0.7937	155.161
YBR182C	10.1651	2.04255
YBR189W	7.1511	2087.86
YBR191W	5.0728	1409.37
YBR200W	3.2916	32.9697
YBR202W	1.4638	58.2689
YBR205W	-3.6366	28.5612
YBR208C	-6.0019	42.8897
YBR213W	11.2396	2.6457
YBR218C	2.7026	422.407
YBR221C	-0.4122	339.193
YBR223C	0.5802	1.25972

YBR248C	-0.3333	294.13
YBR249C	-2.7532	1159.57
YBR252W	1.0119	80.7741
YBR256C	3.1262	60.6548
YBR299W	-0.548	2.77928
YCL017C	1.0595	122.666
YCL018W	-2.114	105.286
YCL035C	6.0851	309.45
YCL043C	2.7162	452.389
YCR008W	3.9709	0.705542
YCR012W	0.1311	16227.3
YCR031C	2.7551	1171.43
YCR053W	6.8393	1292.44
YCR060W	9.8487	23.8212
YCR065W	5.5751	6.97043
YCR067C	3.4547	11.9708
YCR083W	3.4541	38.2323
YCR088W	3.4989	360.96
YDL004W	2.3531	66.7286
YDL007W	2.1028	54.6195
YDL010W	7.4856	8.68243
YDL014W	4.495	518.774
YDL022W	-0.059	699.126
YDL029W	2.1658	76.0204
YDL042C	2.0258	15.3787
YDL045C	0.9625	1.44459
YDL047W	-2.3026	51.9416
YDL056W	6.6455	6.28672
YDL064W	4.4176	108.886
YDL066W	-1.3815	570.686
YDL075W	5.382	1713.65
YDL080C	-0.5246	96.5199
YDL084W	-4.4254	361.409
YDL101C	2.1675	8.69694
YDL102W	-0.829	42.7078
YDL111C	2.4531	45.6747
YDL125C	5.4931	288.451
YDL126C	1.8112	816.452
YDL134C	-2.298	68.8346
YDL137W	1.0021	271.207
YDL143W	1.5998	247.171
YDL160C	-5.0085	210.409
YDL161W	8.319	43.2754
YDL164C	-0.7068	13.442
YDL175C	9.9313	2.26358
YDL188C	-2.2073	30.2855
YDL190C	7.813	51.0141
YDL192W	1.6922	975.554
YDL209C	3.114	6.68938
YDL220C	0.4062	3.19101
YDL229W	-0.9999	2319.31
YDL235C	6.2575	70.4419
YDL244W	3.5835	0.0340762
YDL246C	-1.0934	0.871956
YDR002W	2.6901	678.589
YDR009W	0.1163	1.51837
YDR012W	4.0584	2348.69
YDR021W	-4.9482	9.54011
YDR023W	-2.4881	940.122
YDR035W	-2.7467	360.789

YDR037W	-0.4169	993.372
YDR045C	10.8907	46.7854
YDR047W	-2.3822	44.9743
YDR050C	0.4689	7648.2
YDR059C	4.9411	33.5962
YDR064W	9.4071	1282.42
YDR079C-A	7.7727	5.12155
YDR091C	3.0168	316.792
YDR092W	4.7035	100.635
YDR101C	-2.8115	216.5
YDR129C	7.8603	472.857
YDR139C	4.0511	23.3691
YDR148C	1.5761	78.4075
YDR155C	4.2269	2661.18
YDR158W	-1.0487	1136.45
YDR162C	3.7223	6.71654
YDR165w	6.8181	74.1083
YDR170C	8.3032	72.4936
YDR172W	1.3678	668.476
YDR177W	6.4067	99.8597
YDR189W	3.7039	76.4331
YDR194C	0.6464	146.562
YDR212W	1.4502	94.7855
YDR224C	10.4591	2286.88
YDR225W	9.6808	375.906
YDR226W	0.8868	2077.81
YDR227W	11.1358	21.5249
YDR232W	2.4461	107.722
YDR256C	-3.9132	25.152
YDR258C	0.9305	140.235
YDR267C	-1.1097	10.8775
YDR280W	1.1666	58.0028
YDR341C	6.2385	249.439
YDR353W	1.1103	1162.54
YDR373W	8.825	28.0649
YDR388W	4.7643	172.258
YDR394W	0.1336	94.8337
YDR404C	1.7082	142.956
YDR419W	3.3603	1.20259
YDR424C	3.8218	30.4752
YDR428C	7.3269	3.58131
YDR432W	3.9149	1048.5
YDR436W	-1.7077	2.99385
YDR440W	2.9637	2.46641
YDR441C	2.4501	16.9421
YDR453C	1.212	75.0941
YDR454C	2.01	487.711
YDR471W	5.6495	2486.93
YDR483W	-3.7438	92.8076
YDR487C	4.0849	307.045
YDR500C	10.1998	1219.56
YDR510W	3.3329	315.683
YDR529C	8.4011	357.09
YDR533C	-1.9978	113.694
YEL009C	11.1691	13.9049
YEL011W	4.0815	15.7734
YEL012W	4.8702	19.9435
YEL020W-A	10.7536	132.617
YEL021W	0.4965	176.548
YEL022W	7.1568	53.7306

YEL024W	2.5365	78.496
YEL026W	2.8536	1229.35
YEL030W	1.3507	85.9986
YEL032W	1.5671	26.7835
YEL034W	3.436	1483.72
YEL038W	4.4478	24.467
YEL039C	8.2361	37.1947
YEL048C	9.9535	11.5731
YEL066W	5.3489	14.4008
YER003C	-1.4223	352.45
YER009W	2.5646	137.695
YER012W	2.0377	144.378
YER013W	0.3859	2.8734
YER017C	1.9908	36.4195
YER042W	-0.8888	69.5493
YER043C	1.0103	2392.63
YER047C	1.3637	0.493641
YER057C	1.2933	301.103
YER062C	0.7243	298.183
YER066W	3.2839	0.111255
YER069W	-1.2895	245.646
YER070W	-10.1403	5026.97
YER094C	1.6401	88.3292
YER095W	9.9889	101.76
YER099C	2.9648	35.6923
YER103W	-0.5224	475.352
YER133W	-1.7026	576.43
YER136W	-0.5619	342.874
YER148W	3.7738	131.529
YER165W	3.3443	2162.18
YER172C	1.4568	15.5966
YER175C	5.2404	19.2276
YFL005W	2.0586	94.0103
YFL014W	9.9297	928.52
YFL017C	4.0596	11.8708
YFL017W-A	4.0912	47.0094
YFL018C	2.0343	362.67
YFL022C	-1.282	283.554
YFL030W	2.9159	5.46084
YFL037W	-4.1555	415.383
YFL038C	0.659	188.563
YFL039C	1.7752	2103.33
YFL041W	-7.1752	60.824
YFL058W	3.5719	2.70896
YFL059W	7.8287	3.61184
YFR033C	9.6725	111.438
YFR034C	11.0531	1.0011
YFR047C	3.7378	32.486
YGL004C	-0.8912	15.7949
YGL008C	1.668	2991.27
YGL011C	0.7341	396.011
YGL014W	8.3713	57.8456
YGL018C	10.5046	18.4032
YGL030W	3.8055	914.928
YGL031C	8.2906	3057.16
YGL037C	-0.9344	331.733
YGL043W	6.7275	80.6002
YGL044C	3.9441	7.68374
YGL047W	2.777	11.4056
YGL048C	2.5639	220.889

YGL058W	4.1091	87.7516
YGL068W	5.1863	228.958
YGL070C	5.2687	160.394
YGL076C	1.5324	2050.06
YGL087C	5.3635	114.113
YGL103W	5.0558	2106.56
YGL106W	8.5173	160.432
YGL115W	0.0232	122.508
YGL120C	0.3281	187.387
YGL123W	4.3153	2665.16
YGL130W	2.2616	25.923
YGL134W	8.3341	1.42577
YGL137W	0.5425	403.144
YGL147C	3.2703	2098.92
YGL163C	3.9807	2.41965
YGL187C	4.8766	199.819
YGL190C	1.126	30.1006
YGL194C	1.0867	20.0411
YGL201C	1.4049	46.8608
YGL202W	-1.7	767.593
YGL207W	-2.5264	216.075
YGL212W	7.2851	23.2673
YGL213C	0.672	33.1617
YGL224C	0.3908	9.43037
YGL234W	2.4095	750.351
YGL236C	2.5143	3.1307
YGL240W	1.0547	3.46683
YGL256W	-1.1489	5.60648
YGR027C	7.8617	2863.8
YGR029W	8.6262	4.09781
YGR033C	5.7893	13.8678
YGR034W	4.6622	1929.87
YGR037C	8.0365	1218.54
YGR043C	-0.4876	22.0401
YGR061C	-3.6879	686.328
YGR085C	1.8564	1658.86
YGR087C	-0.6692	397.762
YGR095C	0.088	43.6836
YGR116W	11.1014	129.738
YGR123C	4.5223	96.9791
YGR133W	6.3884	10.7352
YGR135W	1.8069	265.482
YGR144W	3.9036	0.108126
YGR148C	8.2906	2855.23
YGR163W	1.6453	22.5757
YGR173W	-0.825	242.112
YGR183C	11.2566	65.2568
YGR192C	-1.5477	22362.9
YGR195W	2.4981	49.1858
YGR202C	2.9026	42.023
YGR203W	6.6795	35.2322
YGR205W	-0.4618	43.7042
YGR207C	-2.712	148.93
YGR209C	3.234	884.475
YGR214W	3.0662	928.262
YGR232W	6.9772	33.8609
YGR234W	1.7005	1097.78
YGR240C	-4.2303	1854.8
YGR253C	1.9932	492.993
YGR254W	1.0479	7489.28

YGR256W	0.485	100.13
YGR267C	2.5803	86.4847
YGR270W	1.7974	5.4237
YGR287C	-0.6028	0.806602
YGR292W	-0.3093	3.3114
YHL001W	6.6657	1788.19
YHL011C	2.5476	185.252
YHL015W	4.2356	1633.27
YHL033C	2.725	4672.3
YHR005C-A	10.7377	119.028
YHR008C	3.4378	334.695
YHR018C	4.5025	368.252
YHR019C	-0.4857	440.577
YHR024C	2.3255	106.063
YHR029C	1.7716	36.6605
YHR030C	1.6668	55.4051
YHR042W	0.8498	226.06
YHR057C	2.7991	2.44574
YHR079C	5.4592	0.133671
YHR087W	5.9571	236.325
YHR091C	0.8967	1.47712
YHR102W	4.603	7.27913
YHR106W	1.1149	85.2462
YHR117W	8.7172	48.216
YHR128W	1.6572	280.163
YHR135C	4.6806	73.2926
YHR165C	-1.2831	5.56555
YHR171W	-0.9271	0.423883
YHR174W	0.2272	24556.4
YHR179W	-2.0611	723.209
YHR183W	0.0975	3864.33
YHR190W	8.2103	62.4751
YHR201C	1.1158	79.8622
YHR210C	-7.6393	0.768614
YIL008W	2.643	31.8863
YIL021W	1.2717	142.879
YIL035C	1.2357	94.4306
YIL051C	1.2483	823.751
YIL053W	0.8613	3124.52
YIL066C	-7.5636	23.6016
YIL075C	4.9661	208.8
YIL078W	-2.0808	667.795
YIL094C	-2.085	217.064
YIL105C	3.3001	42.8581
YIL109C	9.1266	129.215
YIL113W	4.2607	1.67773
YIL125W	3.1592	185.385
YIL133C	2.358	1408.67
YIL142W	1.3557	119.228
YIL145C	1.5603	59.4623
YIL160C	-4.07	76.1767
YIL162W	-10.2001	12.0181
YIR034C	-0.9645	350.914
YIR035C	-1.8855	10.3643
YIR036C	-1.8641	20.9165
YJL026W	4.2264	2305.16
YJL034W	1.8643	523.3
YJL050W	4.0415	80.0777
YJL052W	-1.5477	2791.99
YJL060W	5.2276	38.8452

YJL068C	-2.3637	19.0827
YJL088W	-2.9994	360.626
YJL111W	2.5961	97.016
YJL121C	1.492	207.863
YJL126W	-1.0834	2.47396
YJL153C	-1.4053	41.5913
YJL155C	0.8229	10.0684
YJL164C	4.9274	34.3229
YJL166W	11.1742	71.5182
YJL167W	7.2464	258.765
YJL177W	3.576	1347.95
YJL190C	5.9293	1318.15
YJL191W	0.1672	1106.18
YJL200C	-8.6531	538.018
YJL216C	-0.548	0.00657676
YJR007W	3.2749	451.725
YJR009C	-1.4954	7517.24
YJR010W	0.3311	155.917
YJR045C	1.4164	709.034
YJR047C	3.436	150.374
YJR048W	4.7966	105.462
YJR064W	1.8359	143.185
YJR066W	8.6479	5.08099
YJR069C	3.8776	123.907
YJR074W	1.0376	13.0418
YJR104C	0.0346	2358.13
YJR121W	-0.6009	921.285
YJR123W	5.5692	3474.98
YJR125C	8.4208	23.5293
YJR131W	1.9211	29.2285
YJR139C	2.7806	884.832
YJR153W	-5.5383	2.95176
YJR156C	3.5835	0.422481
YJR159W	-1.0934	0.867586
YKL006W	6.6657	2477.55
YKL012W	8.7915	0.452641
YKL013C	5.9914	134.039
YKL024C	1.3408	134.706
YKL056C	3.4244	2077.7
YKL058W	10.1341	49.9801
YKL067W	2.5172	417.752
YKL069W	2.3385	38.3051
YKL081W	5.6236	1455.83
YKL085W	-1.0604	409.074
YKL091C	1.4361	13.0354
YKL127W	-0.3042	91.0137
YKL135C	8.2564	40.6867
YKL144C	3.9174	3.09982
YKL145W	1.3699	290.209
YKL152C	1.8643	6666.15
YKL166C	4.8699	46.3585
YKL180W	3.576	1687.66
YKL181W	2.5124	158.827
YKL182W	-6.6084	1183.02
YKL190W	8.8767	88.2138
YKL194C	-2.0071	3.36176
YKL196C	3.6957	102.413
YKL203C	8.3492	4.90972
YKL210W	-0.4473	633.381
YKL216W	-2.2372	1871.16

YKL218C	1.4764	7.72838
YKR002W	4.4041	30.7336
YKR008W	9.4099	88.6484
YKR014C	0.5463	228.153
YKR043C	-0.2899	242.289
YKR049C	2.7961	6.60385
YKR068C	2.8875	29.3329
YKR070W	4.7008	17.8229
YKR080W	2.5689	256.671
YKR084C	2.2686	39.1371
YLL009C	9.2947	218.418
YLL010C	0.9762	33.1801
YLL013C	8.4475	46.338
YLL024C	-1.008	7983.67
YLL026W	1.0264	720.989
YLL036C	1.9973	34.6824
YLL039C	3.786	178.551
YLL041C	2.4278	91.8253
YLL045C	4.1635	2793.07
YLL050C	4.2986	669.374
YLL053C	4.9339	6.00849
YLL060C	6.1874	5.09333
YLR008C	9.8463	34.9584
YLR011W	1.8878	1.04566
YLR027C	-0.9273	376.176
YLR028C	1.0142	233.731
YLR029C	5.1803	1983.66
YLR043C	3.5853	714.969
YLR044C	-0.4359	18666.
YLR045C	8.4187	23.4758
YLR048W	3.0662	2397.32
YLR060W	0.5459	661.572
YLR075W	2.3139	6398.89
YLR080W	17.9301	2.50967
YLR089C	5.995	45.1789
YLR093C	3.3827	14.3584
YLR109W	0.4736	9381.19
YLR113W	4.2938	121.525
YLR115W	2.9827	14.3785
YLR134W	-0.4359	683.546
YLR153C	0.8474	1079.05
YLR163C	2.2214	46.2315
YLR167W	10.1446	2900.96
YLR170C	5.534	3.71406
YLR172C	4.0674	97.5438
YLR174W	-1.3815	18.7846
YLR175W	-0.2933	412.152
YLR180W	4.9291	1716.35
YLR185W	10.1998	1792.94
YLR191W	3.5725	4.57722
YLR195C	3.3047	32.5137
YLR216C	1.9561	1892.13
YLR229C	1.0122	87.3999
YLR244C	-3.4694	325.817
YLR245C	3.4296	36.0812
YLR249W	1.2074	6949.26
YLR259C	7.1197	778.948
YLR274W	1.4779	17.9329
YLR286C	-2.2539	41.3226
YLR293C	0.4683	445.946

YLR300W	0.9048	122.257
YLR303W	0.0034	579.245
YLR304C	-9.2044	594.903
YLR306W	4.4077	6.70155
YLR335W	4.4608	87.2841
YLR340W	1.1844	1872.34
YLR344W	4.6833	1964.08
YLR347C	6.8491	368.883
YLR354C	-0.4876	1024.81
YLR359W	7.025	149.493
YLR367W	5.9293	1666.33
YLR370C	11.0922	311.38
YLR377C	1.0244	5.92562
YLR397C	1.7007	32.5415
YLR398C	2.5571	52.445
YLR406C	5.382	1678.32
YLR410W	-0.5469	90.0719
YLR433C	-1.8925	51.2835
YLR438C-A	3.5557	216.307
YLR438W	0.278	260.521
YLR442C	1.2997	11.8227
YLR449W	0.8482	139.048
YML001W	0.3998	115.631
YML016C	-1.7026	40.9598
YML021C	1.1241	3.34709
YML022W	2.593	289.729
YML028W	1.212	4859.15
YML035C	6.0567	61.5968
YML051W	3.7309	1.98466
YML054C	3.4014	15.2425
YML057W	-1.8925	97.2577
YML074C	0.9334	265.456
YML078W	4.4102	205.793
YML085C	-5.8089	290.938
YML092C	0.6331	356.533
YML106W	2.9776	333.496
YML108W	3.8166	17.5507
YML120C	1.9393	153.919
YML124C	-5.8089	151.854
YML126C	3.308	899.454
YMR020W	3.0219	4.85036
YMR022W	5.1111	17.7816
YMR037C	9.7138	24.8442
YMR038C	-0.577	374.531
YMR043W	10.4603	47.8427
YMR047C	2.2833	6.76737
YMR058W	-7.1518	333.917
YMR074C	9.5595	80.0307
YMR079W	1.9015	554.317
YMR089C	2.3326	53.7914
YMR092C	1.8944	189.091
YMR096W	7.7611	9.92266
YMR099C	0.2832	176.078
YMR105C	0.1737	334.504
YMR116C	-0.8489	2895.19
YMR117C	9.21	44.0465
YMR120C	1.0142	1933.92
YMR121C	5.1803	965.777
YMR145C	-0.5154	100.443
YMR159C	11.125	1.26567

YMR170C	-0.5387	19.1733
YMR174C	11.2339	16.2527
YMR186W	-0.2417	1221.64
YMR197C	10.1211	6.11326
YMR205C	-0.0116	1909.44
YMR207C	-4.4752	2.64732
YMR213W	10.4612	1.47429
YMR226C	-0.8242	445.601
YMR228W	-1.3863	5.38305
YMR230W	6.6405	1059.09
YMR239C	6.2211	30.1418
YMR260C	4.6727	483.211
YMR268C	3.7684	1.16109
YMR271C	3.2906	16.67
YMR289W	1.048	1.92443
YMR297W	0.7286	100.649
YMR303C	-1.0236	1010.36
YMR308C	7.2545	124.448
YMR314W	1.1781	342.913
YMR318C	-1.1941	262.034
YMR323W	0.8949	7.74468
YNL001W	4.4297	72.412
YNL007C	2.8566	524.232
YNL009W	-1.3778	26.0446
YNL014W	0.942	120.879
YNL036W	2.4272	66.1853
YNL037C	-1.1784	320.685
YNL045W	-3.7481	60.3555
YNL049C	9.0996	30.387
YNL053W	4.2607	1.95607
YNL067W	3.2703	4455.56
YNL068C	6.3582	5.61515
YNL069C	2.3276	1559.73
YNL088W	0.3395	32.4481
YNL090W	0.8832	43.9916
YNL097C	7.6999	9.68068
YNL098C	1.1994	235.247
YNL102W	-0.6976	10.2802
YNL108C	0.7591	54.0034
YNL112W	1.0704	694.894
YNL135C	0.8527	644.86
YNL138W	1.2389	253.807
YNL147W	3.0647	101.039
YNL154C	4.3732	94.077
YNL168C	5.5601	52.5956
YNL178W	4.5885	2257.9
YNL185C	5.9058	55.2099
YNL189W	8.2339	80.473
YNL200C	3.3903	18.2007
YNL209W	-0.9999	2791.
YNL220W	2.3975	522.195
YNL229C	6.0194	60.0454
YNL231C	-0.7993	191.465
YNL238W	-6.0943	38.853
YNL241C	-1.6397	269.011
YNL244C	4.4064	849.754
YNL259C	4.7384	66.274
YNL264C	3.4101	13.713
YNL271C	4.944	7.8627
YNL290W	3.1683	58.0514

YNL298W	2.0173	10.31
YNL302C	5.8666	1632.58
YNL328C	10.2809	6.07161
YNL329C	2.4461	10.4626
YNL330C	1.0007	43.269
YNL332W	3.5835	0.0349222
YNL333W	7.8287	3.54696
YNR011C	-4.9837	0.617118
YNR016C	3.0797	579.516
YNR026C	1.7128	28.415
YNR032C-A	3.2736	10.069
YNR032W	-2.1799	5.73767
YNR034W-A	3.8416	106.248
YNR043W	5.7203	261.461
YNR051C	1.1423	116.685
YNR071C	-7.6393	1.95993
YOL005C	6.0222	146.628
YOL006C	6.9403	51.4256
YOL010W	-1.2987	65.8938
YOL021C	5.8154	47.0605
YOL023W	-2.1724	32.0163
YOL027C	5.6669	95.937
YOL038W	1.2207	230.316
YOL040C	8.147	3408.96
YOL049W	5.6783	30.1268
YOL059W	-0.059	302.542
YOL064C	1.594	131.364
YOL068C	2.0258	1.62548
YOL078W	6.9294	3.65683
YOL086C	-1.0283	8176.9
YOL094C	3.4266	40.9132
YOL097C	1.5708	311.168
YOL113W	3.0991	3.351
YOL123W	4.156	89.152
YOL127W	5.2293	2403.2
YOL133W	12.1753	26.3561
YOL135C	10.6518	10.9465
YOL139C	-0.2918	1011.31
YOL141W	-1.1199	9.33588
YOL143C	1.0475	221.646
YOL157C	-0.86	2.17365
YOL164W	1.7773	1.06822
YOR001W	1.8091	23.4095
YOR026W	-1.5467	19.8935
YOR046C	-4.2987	177.327
YOR061W	3.913	119.237
YOR064C	6.8114	0.736488
YOR065W	2.601	124.08
YOR070C	5.3814	0.417977
YOR074C	2.7926	24.8048
YOR084W	4.7908	49.3275
YOR089C	4.6476	185.692
YOR094W	1.3021	58.8164
YOR095C	2.2194	88.2258
YOR101W	0.9807	21.189
YOR106W	8.1261	1.45832
YOR117W	0.2667	82.2322
YOR122C	3.8749	956.254
YOR126C	0.5244	6.80075
YOR136W	-1.8682	554.292

YOR141C	5.7893	19.4344
YOR142W	1.1332	185.808
YOR143C	1.9006	4.56432
YOR151C	7.1562	292.109
YOR168W	-3.1832	421.432
YOR185C	0.4683	364.169
YOR187W	-5.6281	299.111
YOR190W	2.1451	1.27784
YOR194C	4.497	6.35556
YOR204W	0.7119	291.535
YOR207C	7.1966	55.8129
YOR210W	8.8618	276.424
YOR236W	-1.6659	23.7419
YOR244W	4.7	9.54742
YOR250C	5.9245	7.08275
YOR251C	-0.7121	29.4825
YOR257W	9.2483	6.20988
YOR259C	1.9656	523.555
YOR265W	9.6309	86.1063
YOR285W	4.6837	269.134
YOR288C	3.0386	11.5768
YOR298C-A	7.5297	1206.06
YOR339C	5.7915	6.60514
YOR357C	5.5868	14.5042
YOR358W	10.3933	1.05718
YOR359W	9.9163	33.9793
YOR362C	1.3466	309.283
YOR369C	3.9152	1673.42
YOR388C	2.2979	9.48239
YPL001W	4.056	7.55805
YPL020C	4.4863	0.71753
YPL031C	4.5631	40.8447
YPL036W	1.898	846.945
YPL046C	6.9153	1.47999
YPL059W	4.869	94.9213
YPL063W	1.2478	110.712
YPL065W	8.5299	2.90908
YPL069C	7.5911	14.6392
YPL079W	5.0728	1226.81
YPL081W	5.1835	2113.66
YPL084W	11.3183	54.3045
YPL089C	10.2731	5.40067
YPL091W	1.5392	227.969
YPL106C	-0.7937	1959.27
YPL111W	-4.1692	505.447
YPL119C	0.3987	23.8397
YPL131W	3.4522	2121.97
YPL141C	5.1565	3.09935
YPL153C	-0.0408	11.0961
YPL154C	2.3562	225.841
YPL171C	-2.0611	17.1141
YPL179W	-1.6246	0.303096
YPL198W	1.3015	1573.57
YPL203W	4.8699	21.4701
YPL204W	4.051	77.1151
YPL214C	0.8214	0.659275
YPL218W	1.3253	185.927
YPL228W	-1.8956	21.6122
YPL231W	-0.8648	1075.99
YPL235W	7.1904	89.1039

YPL239W	7.4869	160.738
YPL240C	-0.2417	2116.84
YPL248C	9.9665	32.2668
YPL252C	3.1114	81.7822
YPL266W	0.4859	24.4823
YPR016C	-0.4823	308.602
YPR024W	2.5102	50.6963
YPR031W	7.9917	0.541798
YPR032W	2.7134	16.546
YPR034W	6.4508	70.5072
YPR035W	2.6307	2388.65
YPR036W	9.3284	421.494
YPR041W	6.5376	546.004
YPR052C	8.1498	437.711
YPR060C	4.5061	188.57
YPR062W	4.3269	256.209
YPR069C	-0.0954	420.198
YPR073C	1.9498	47.104
YPR074C	-1.914	1750.43
YPR081C	0.9306	8.10728
YPR082C	2.4023	4.67793
YPR088C	0.8827	24.3282
YPR094W	2.5922	2.59199
YPR102C	1.8564	842.123
YPR108W	8.1406	167.577
YPR160W	0.364	91.1769
YPR165W	0.0519	255.524
YPR167C	1.8164	5.83235
YPR173C	1.985	48.1596
YPR176C	6.4092	1.41831
YPR182W	3.3638	55.645
YPR187W	6.0106	287.356
YPR189W	8.9503	51.4876
YPR191W	3.0268	259.89
YPR193C	5.3489	0.140777
