# Supplementary material for: Proteome Folding Kinetics Is Limited by Protein Halflife
Source: PLoS One. 2014 Nov 13;9(11):e112701. doi: 10.1371/journal.pone.0112701 (PMC4231061; doi:10.1371/journal.pone.0112701)
Supplement: Table S3 — Dataset of folding time and halflife for Yeast proteome. First column reports Open Reading Frame as reported in YRC [22]; second column reports halflife (in minutes) from O′Shea et al. [27]; third column reports where is the folding speed for the slowest folding domain in the units of . (PDF) [file pone.0112701.s003.pdf]

Protein Name	half life	lnk_f
YAL005C	77.	-1.008
YAL036C	57.	-0.8232
YAL054C	29.	0.8474
YAR007C	35.	0.0176
YAR015W	77.	0.5259
YBL002W	300.	10.3077
YBL003C	167.	9.6808
YBL007C	41.	3.6146
YBL016W	43.	5.5994
YBL023C	124.	1.5802
YBL036C	300.	0.8874
YBL045C	291.	2.6272
YBL052C	7.	6.6829
YBL068W	116.	0.6985
YBL075C	59.	-0.4601
YBL078C	289.	4.32
YBL099W	49.	-1.2054
YBR006W	31.	-0.9123
YBR011C	883.	-2.7881
YBR025C	136.	8.6771
YBR034C	43.	-3.9063
YBR082C	12.	4.9411
YBR087W	138.	8.8626
YBR089C-A	103.	8.0965
YBR097W	2.	3.9846
YBR121C	44.	0.764
YBR143C	62.	3.2399
YBR160W	300.	4.2391
YBR164C	300.	1.518
YBR169C	5252.	-0.7937
YBR189W	29.	7.1511
YBR200W	19.	3.2916
YBR205W	17.	-3.6366
YBR208C	45.	-6.0019
YBR218C	40.	2.7026
YBR221C	72.	-0.4122
YBR248C	56.	-0.3333
YBR249C	71.	-2.7532
YBR252W	300.	1.0119
YBR256C	280.	3.1262
YBR299W	42.	-0.548
YCL035C	300.	6.0851
YCL043C	8.	2.7162
YCR008W	12.	3.9709
YCR012W	199.	0.1311
YCR031C	40.	2.7551
YCR053W	148.	6.8393
YCR060W	47.	9.8487
YCR065W	15.	5.5751
YCR067C	62.	3.4547
YCR083W	175.	3.4541
YCR088W	18.	3.4989
YDL007W	17.	2.1028
YDL010W	2.	7.4856
YDL022W	250.	-0.059
YDL029W	300.	2.1658
YDL042C	21.	2.0258
YDL045C	1197.	0.9625
YDL047W	34.	-2.3026
YDL056W	20.	6.6455

YDL064W	86.	4.4176
YDL080C	33.	-0.5246
YDL084W	17.	-4.4254
YDL101C	102.	2.1675
YDL111C	300.	2.4531
YDL125C	267.	5.4931
YDL126C	34.	1.8112
YDL134C	45.	-2.298
YDL137W	113.	1.0021
YDL143W	125.	1.5998
YDL160C	176.	-5.0085
YDL161W	39.	8.319
YDL175C	30.	9.9313
YDL188C	61.	-2.2073
YDL190C	300.	7.813
YDL192W	300.	1.6922
YDL209C	75.	3.114
YDL220C	42.	0.4062
YDL229W	149.	-0.9999
YDL235C	107.	6.2575
YDR009W	18.	0.1163
YDR012W	300.	4.0584
YDR021W	46.	-4.9482
YDR035W	61.	-2.7467
YDR047W	845.	-2.3822
YDR050C	96.	0.4689
YDR059C	77.	4.9411
YDR091C	38.	3.0168
YDR092W	300.	4.7035
YDR101C	96.	-2.8115
YDR129C	96.	7.8603
YDR148C	51.	1.5761
YDR155C	300.	4.2269
YDR158W	105.	-1.0487
YDR165W	26.	6.8181
YDR170C	65.	8.3032
YDR177W	45.	6.4067
YDR189W	32.	3.7039
YDR194C	162.	0.6464
YDR224C	300.	10.4591
YDR226W	217.	0.8868
YDR256C	31.	-3.9132
YDR258C	57.	0.9305
YDR267C	97.	-1.1097
YDR373W	34.	8.825
YDR388W	41.	4.7643
YDR404C	35.	1.7082
YDR419W	61.	3.3603
YDR424C	10.	3.8218
YDR436W	43.	-1.7077
YDR440W	63.	2.9637
YDR441C	28.	2.4501
YDR453C	149.	1.212
YDR454C	300.	2.01
YDR483W	2.	-3.7438
YDR487C	20.	4.0849
YDR500C	53.	10.1998
YDR510W	7.	3.3329
YDR533C	300.	-1.9978
YEL009C	5.	11.1691

YEL012W	87.	4.8702
YEL022W	16.	7.1568
YEL032W	31.	1.5671
YEL039C	236.	8.2361
YEL048C	607.	9.9535
YEL066W	3.	5.3489
YER003C	29.	-1.4223
YER012W	1131.	2.0377
YER013W	45.	0.3859
YER017C	45.	1.9908
YER042W	145.	-0.8888
YER047C	34.	1.3637
YER057C	300.	1.2933
YER062C	300.	0.7243
YER070W	77.	-10.1403
YER095W	60.	9.9889
YER099C	300.	2.9648
YER103W	300.	-0.5224
YER133W	61.	-1.7026
YER136W	300.	-0.5619
YER172C	8.	1.4568
YER175C	300.	5.2404
YFL017C	25.	4.0596
YFL017W-A	297.	4.0912
YFL041W	9.	-7.1752
YFR033C	146.	9.6725
YFR047C	239.	3.7378
YGL004C	53.	-0.8912
YGL008C	45.	1.668
YGL011C	73.	0.7341
YGL014W	22.	8.3713
YGL018C	46.	10.5046
YGL030W	146.	3.8055
YGL031C	113.	8.2906
YGL037C	300.	-0.9344
YGL043W	184.	6.7275
YGL044C	71.	3.9441
YGL047W	300.	2.777
YGL048C	36.	2.5639
YGL058W	114.	4.1091
YGL068W	59.	5.1863
YGL070C	87.	5.2687
YGL076C	113.	1.5324
YGL087C	51.	5.3635
YGL115W	300.	0.0232
YGL120C	32.	0.3281
YGL130W	3.	2.2616
YGL134W	10.	8.3341
YGL137W	53.	0.5425
YGL147C	53.	3.2703
YGL163C	14.	3.9807
YGL190C	80.	1.126
YGL194C	125.	1.0867
YGL201C	21.	1.4049
YGL202W	59.	-1.7
YGL207W	26.	-2.5264
YGL212W	61.	7.2851
YGL213C	258.	0.672
YGL234W	162.	2.4095
YGL240W	17.	1.0547

YGL256W	300.	-1.1489
YGR027C	82.	7.8617
YGR033C	26.	5.7893
YGR034W	300.	4.6622
YGR037C	300.	8.0365
YGR043C	61.	-0.4876
YGR061C	33.	-3.6879
YGR085C	300.	1.8564
YGR095C	101.	0.088
YGR116W	36.	11.1014
YGR123C	125.	4.5223
YGR133W	82.	6.3884
YGR135W	47.	1.8069
YGR148C	54.	8.2906
YGR163W	37.	1.6453
YGR173W	150.	-0.825
YGR183C	2048.	11.2566
YGR195W	300.	2.4981
YGR202C	26.	2.9026
YGR203W	70.	6.6795
YGR205W	472.	-0.4618
YGR207C	300.	-2.712
YGR214W	300.	3.0662
YGR232W	300.	6.9772
YGR234W	83.	1.7005
YGR253C	300.	1.9932
YGR270W	893.	1.7974
YGR287C	4816.	-0.6028
YGR292W	35.	-0.3093
YHL001W	163.	6.6657
YHR008C	300.	3.4378
YHR024C	53.	2.3255
YHR029C	138.	1.7716
YHR030C	62.	1.6668
YHR042W	61.	0.8498
YHR057C	7.	2.7991
YHR079C	32.	5.4592
YHR087W	45.	5.9571
YHR106W	38.	1.1149
YHR117W	98.	8.7172
YHR135C	14.	4.6806
YHR174W	27.	0.2272
YHR179W	107.	-2.0611
YHR201C	139.	1.1158
YIL008W	86.	2.643
YIL021W	29.	1.2717
YIL035C	62.	1.2357
YIL051C	809.	1.2483
YIL053W	113.	0.8613
YIL066C	64.	-7.5636
YIL075C	400.	4.9661
YIL094C	18.	-2.085
YIL105C	21.	3.3001
YIL125W	85.	3.1592
YIL133C	1308.	2.358
YIL145C	300.	1.5603
YIL160C	30.	-4.07
YIR034C	300.	-0.9645
YIR035C	300.	-1.8855
YIR036C	54.	-1.8641

YJL034W	124.	1.8643
YJL050W	47.	4.0415
YJL052W	85.	-1.5477
YJL060W	32.	5.2276
YJL068C	243.	-2.3637
YJL088W	49.	-2.9994
YJL121C	1797.	1.492
YJL153C	38.	-1.4053
YJL155C	41.	0.8229
YJL164C	300.	4.9274
YJL166W	7.	11.1742
YJL167W	13.	7.2464
YJL177W	21.	3.576
YJL190C	78.	5.9293
YJL191W	300.	0.1672
YJL200C	34.	-8.6531
YJR007W	42.	3.2749
YJR010W	44.	0.3311
YJR047C	12.	3.436
YJR048W	61.	4.7966
YJR066W	5.	8.6479
YJR069C	51.	3.8776
YJR074W	29.	1.0376
YJR104C	300.	0.0346
YJR121W	6627.	-0.6009
YJR131W	84.	1.9211
YJR139C	56.	2.7806
YJR156C	3.	3.5835
YKL006W	300.	6.6657
YKL013C	300.	5.9914
YKL024C	329.	1.3408
YKL056C	166.	3.4244
YKL058W	170.	10.1341
YKL067W	300.	2.5172
YKL069W	25.	2.3385
YKL081W	105.	5.6236
YKL085W	167.	-1.0604
YKL091C	170.	1.4361
YKL127W	23.	-0.3042
YKL135C	71.	8.2564
YKL144C	142.	3.9174
YKL145W	9.	1.3699
YKL152C	300.	1.8643
YKL166C	24.	4.8699
YKL181W	44.	2.5124
YKL182W	300.	-6.6084
YKL194C	134.	-2.0071
YKL210W	114.	-0.4473
YKL216W	203.	-2.2372
YKR002W	23.	4.4041
YKR008W	55.	9.4099
YKR014C	773.	0.5463
YKR043C	300.	-0.2899
YKR068C	44.	2.8875
YKR070W	71.	4.7008
YKR080W	197.	2.5689
YKR084C	43.	2.2686
YLL010C	758.	0.9762
YLL013C	326.	8.4475
YLL026W	61.	1.0264

YLL036C	253.	1.9973
YLL039C	13.	3.786
YLL045C	80.	4.1635
YLL050C	300.	4.2986
YLL060C	24.	6.1874
YLR011W	84.	1.8878
YLR027C	205.	-0.9273
YLR028C	59.	1.0142
YLR044C	29.	-0.4359
YLR045C	19.	8.4187
YLR060W	44.	0.5459
YLR089C	78.	5.995
YLR093C	46.	3.3827
YLR109W	300.	0.4736
YLR113W	273.	4.2938
YLR115W	12.	2.9827
YLR163C	1961.	2.2214
YLR172C	403.	4.0674
YLR174W	69.	-1.3815
YLR175W	30.	-0.2933
YLR185W	316.	10.1998
YLR191W	44.	3.5725
YLR216C	89.	1.9561
YLR244C	669.	-3.4694
YLR245C	300.	3.4296
YLR249W	191.	1.2074
YLR274W	43.	1.4779
YLR300W	15.	0.9048
YLR304C	72.	-9.2044
YLR335W	21.	4.4608
YLR344W	358.	4.6833
YLR347C	36.	6.8491
YLR354C	136.	-0.4876
YLR367W	181.	5.9293
YLR370C	51.	11.0922
YLR377C	81.	1.0244
YLR398C	40.	2.5571
YLR406C	265.	5.382
YLR433C	32.	-1.8925
YLR449W	11.	0.8482
YML016C	50.	-1.7026
YML021C	36.	1.1241
YML022W	300.	2.593
YML028W	300.	1.212
YML035C	33.	6.0567
YML051W	2856.	3.7309
YML054C	54.	3.4014
YML057W	47.	-1.8925
YML078W	58.	4.4102
YML085C	41.	-5.8089
YML092C	7987.	0.6331
YML108W	59.	3.8166
YML120C	43.	1.9393
YML124C	448.	-5.8089
YML126C	46.	3.308
YMR020W	54.	3.0219
YMR022W	31.	5.1111
YMR037C	2.	9.7138
YMR038C	300.	-0.577
YMR043W	300.	10.4603

YMR058W	46.	-7.1518
YMR074C	300.	9.5595
YMR079W	149.	1.9015
YMR089C	29.	2.3326
YMR105C	43.	0.1737
YMR116C	157.	-0.8489
YMR117C	74.	9.21
YMR120C	39.	1.0142
YMR121C	142.	5.1803
YMR159C	56.	11.125
YMR170C	300.	-0.5387
YMR174C	300.	11.2339
YMR186W	109.	-0.2417
YMR205C	96.	-0.0116
YMR213W	28.	10.4612
YMR226C	47.	-0.8242
YMR228W	36.	-1.3863
YMR230W	1448.	6.6405
YMR239C	103.	6.2211
YMR260C	32.	4.6727
YMR268C	52.	3.7684
YMR271C	32.	3.2906
YMR289W	300.	1.048
YMR297W	13.	0.7286
YMR303C	41.	-1.0236
YMR308C	88.	7.2545
YMR314W	300.	1.1781
YMR318C	57.	-1.1941
YNL001W	106.	4.4297
YNL007C	274.	2.8566
YNL009W	13.	-1.3778
YNL014W	3.	0.942
YNL036W	14.	2.4272
YNL037C	29.	-1.1784
YNL053W	15.	4.2607
YNL067W	300.	3.2703
YNL068C	26.	6.3582
YNL088W	81.	0.3395
YNL097C	49.	7.6999
YNL098C	25.	1.1994
YNL102W	300.	-0.6976
YNL108C	300.	0.7591
YNL135C	196.	0.8527
YNL138W	87.	1.2389
YNL147W	300.	3.0647
YNL154C	18.	4.3732
YNL168C	444.	5.5601
YNL185C	169.	5.9058
YNL189W	39.	8.2339
YNL200C	141.	3.3903
YNL209W	712.	-0.9999
YNL220W	57.	2.3975
YNL229C	39.	6.0194
YNL231C	69.	-0.7993
YNL238W	93.	-6.0943
YNL241C	300.	-1.6397
YNL259C	39.	4.7384
YNL264C	52.	3.4101
YNL271C	79.	4.944
YNL290W	54.	3.1683

YNL298W	105.	2.0173
YNL329C	44.	2.4461
YNL330C	1168.	1.0007
YNR011C	7.	-4.9837
YNR016C	233.	3.0797
YNR026C	61.	1.7128
YNR032W	39.	-2.1799
YNR034W-A	300.	3.8416
YNR043W	65.	5.7203
YNR051C	18.	1.1423
YOL005C	300.	6.0222
YOL006C	35.	6.9403
YOL010W	46.	-1.2987
YOL021C	27.	5.8154
YOL023W	11.	-2.1724
YOL027C	43.	5.6669
YOL038W	300.	1.2207
YOL049W	75.	5.6783
YOL059W	116.	-0.059
YOL064C	31.	1.594
YOL068C	56.	2.0258
YOL078W	11.	6.9294
YOL094C	300.	3.4266
YOL097C	153.	1.5708
YOL113W	21.	3.0991
YOL135C	37.	10.6518
YOL141W	23.	-1.1199
YOL143C	61.	1.0475
YOR001W	19.	1.8091
YOR026W	22.	-1.5467
YOR046C	70.	-4.2987
YOR061W	54.	3.913
YOR065W	161.	2.601
YOR070C	21.	5.3814
YOR084W	63.	4.7908
YOR095C	300.	2.2194
YOR101W	106.	0.9807
YOR106W	13.	8.1261
YOR136W	300.	-1.8682
YOR141C	199.	5.7893
YOR142W	1026.	1.1332
YOR143C	82.	1.9006
YOR168W	25.	-3.1832
YOR207C	25.	7.1966
YOR236W	36.	-1.6659
YOR244W	208.	4.7
YOR250C	9.	5.9245
YOR251C	296.	-0.7121
YOR265W	30.	9.6309
YOR285W	140.	4.6837
YOR288C	20.	3.0386
YOR298C-A	106.	7.5297
YOR357C	29.	5.5868
YOR358W	300.	10.3933
YOR359W	22.	9.9163
YOR362C	53.	1.3466
YPL020C	21.	4.4863
YPL031C	217.	4.5631
YPL046C	5.	6.9153
YPL059W	94.	4.869

YPL063W	39.	1.2478
YPL065W	259.	8.5299
YPL069C	95.	7.5911
YPL079W	84.	5.0728
YPL081W	169.	5.1835
YPL084W	27.	11.3183
YPL089C	32.	10.2731
YPL091W	35.	1.5392
YPL106C	101.	-0.7937
YPL111W	69.	-4.1692
YPL119C	18.	0.3987
YPL153C	2.	-0.0408
YPL198W	82.	1.3015
YPL203W	25.	4.8699
YPL204W	25.	4.051
YPL214C	77.	0.8214
YPL228W	33.	-1.8956
YPL231W	19.	-0.8648
YPL235W	250.	7.1904
YPL239W	25.	7.4869
YPR016C	69.	-0.4823
YPR024W	52.	2.5102
YPR031W	12.	7.9917
YPR032W	46.	2.7134
YPR034W	762.	6.4508
YPR035W	73.	2.6307
YPR036W	223.	9.3284
YPR041W	186.	6.5376
YPR060C	3.	4.5061
YPR062W	300.	4.3269
YPR073C	44.	1.9498
YPR081C	38.	0.9306
YPR102C	71.	1.8564
YPR108W	300.	8.1406
YPR167C	54.	1.8164
YPR173C	57.	1.985
YPR176C	300.	6.4092
YPR187W	300.	6.0106
YPR189W	32.	8.9503
YPR191W	352.	3.0268
